# Interpretable PROTAC Degradation Prediction With Structure‐Informed Deep Ternary Attention Framework

**DOI:** 10.1002/advs.202508138

**Published:** 2025-09-30

**Authors:** Zhenglu Chen, Chunbin Gu, Shuoyan Tan, Xiaorui Wang, Yuquan Li, Mutian He, Ruiqiang Lu, Shijia Sun, Chang‐Yu Hsieh, Xiaojun Yao, Huanxiang Liu, Pheng‐Ann Heng

**Affiliations:** ^1^ School of Pharmacy Lanzhou University Lanzhou 730000 China; ^2^ Department of Computer Science and Engineering The Chinese University of Hong Kong Hong Kong 999077 China; ^3^ Innovation Center of Yangtze River Delta Zhejiang University Jiaxing 314100 China; ^4^ College of Pharmaceutical Sciences Zhejiang University Hangzhou 310058 China; ^5^ College of Chemistry and Chemical Engineering Lanzhou University Lanzhou 730000 China; ^6^ Faculty of Applied Sciences Macao Polytechnic University Macao 999078 China

**Keywords:** deep learning, interpretability, molecular dynamics simulation, proteolysis targeting chimera

## Abstract

Proteolysis Targeting Chimeras (PROTACs) are heterobifunctional ligands bridging Proteins‐Of‐Interest (POIs) and E3 ligases for ubiquitin‐proteasome degradation, promising to target the ‘undruggable’. While PROTAC research primarily relies on costly and time‐consuming wet‐lab experiments, deep learning offers potential to accelerate development and reduce expenses. However, many deep learning methods for PROTAC degradation prediction overlook hierarchical molecular representation and protein structural data, hindering data modeling. Moreover, their black‐box nature hampers interpretability, failing to provide intuitive insights into substructure interactions within the PROTAC system. This study introduces PROTAC‐STAN, a structure‐informed deep ternary attention network (STAN) framework for interpretable PROTAC degradation prediction. PROTAC‐STAN represents PROTAC molecules across atom, molecule, and property hierarchies and incorporates structure information for POIs and E3 ligases via protein language model. Furthermore, it simulates interactions among three entities at substructure levels via a novel ternary attention network tailored for the PROTAC system, providing unprecedented insights into the degradation mechanism. PROTAC‐STAN yields over 10% improvement across multiple metrics compared to the best baselines, while providing significant interpretability through atomic and residue level visualization of molecule and complex. Exploratory evaluations and case studies demonstrate strong real‐world applicability. The excellence of PROTAC‐STAN is anticipated to establish a foundational tool for future PROTAC research.

## Graphical abstract

PROTAC‐STAN, a structure‐informed deep learning framework is presented for interpretable PROTAC degradation prediction. By modeling molecular hierarchies and protein structures, and simulating ternary interactions via a novel attention network, PROTAC‐STAN achieves significant performance gains over baselines. It also offers atomic‐ and residue‐level interpretability, providing valuable mechanistic insights and a promising tool for advancing PROTAC research.

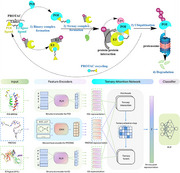

## 1 Introduction

**Figure 1:**
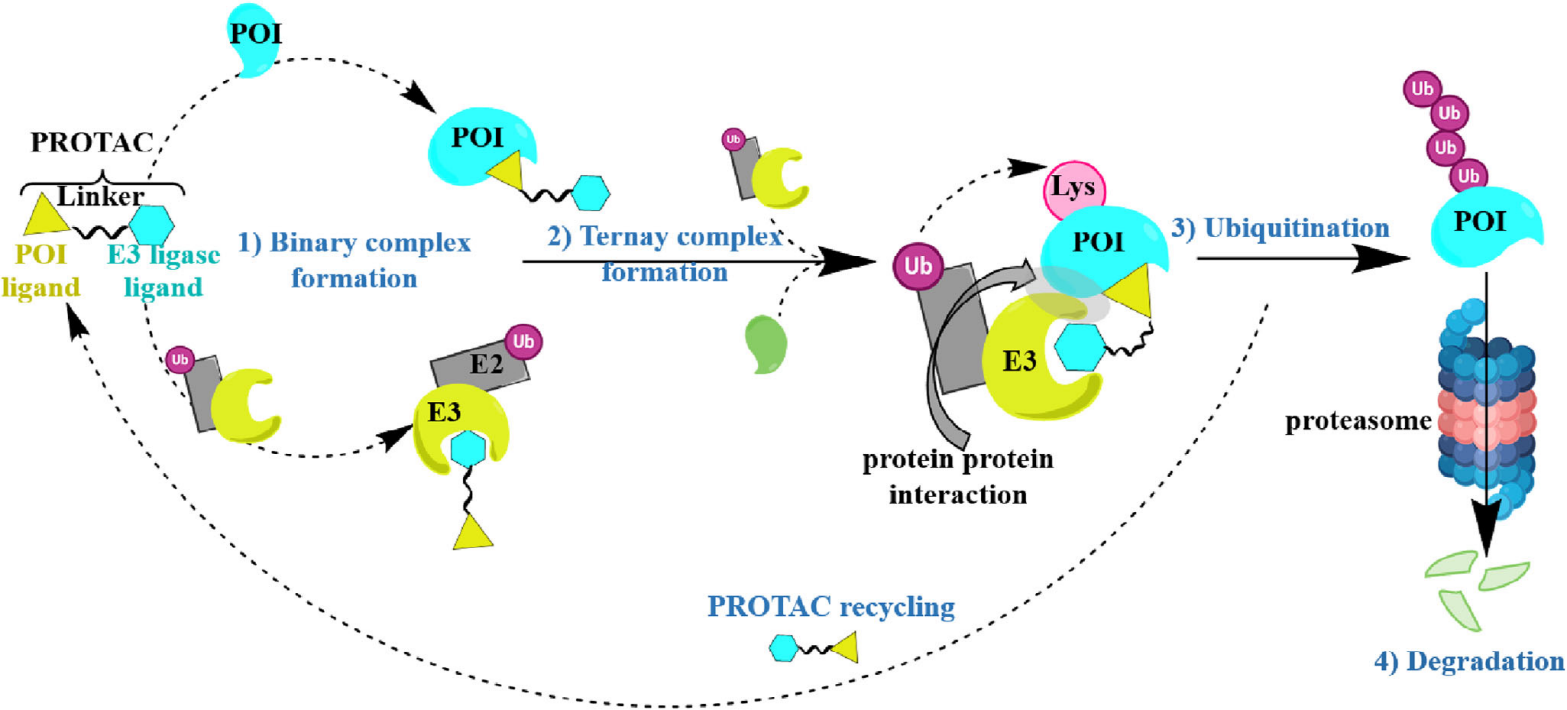
PROTAC‐mediated protein degradation. PROTACs are heterobifunctional molecules designed to induce targeted protein degradation. The figure illustrates the mechanism by which PROTACs function: one end of the PROTAC molecule binds to the target protein, while the other end recruits an E3 ligase. This interaction facilitates the ubiquitination of the target protein, marking it for degradation by the proteasome. The process results in the efficient and selective degradation of the target protein, showcasing the potential of PROTACs as powerful tools for targeted therapy.

Targeted Protein Degradation (TPD) has presented a promising approach to modern drug development in recent years.^[^ ^1^, ^2^, ^3^ ^]^ Unlike traditional drug design methods that typically modulate protein function through inhibition or activation, TPD targets proteins for degradation by marking them as substrates for cellular degradation machinery, thus achieving therapeutic outcomes^[^ ^1^ ^]^. TPD holds the potential to address proteins considered ‘undruggable’ by traditional methods and may mitigate resistance caused by target pocket mutations.^[^ ^2^, ^4^ ^]^ Moreover, it offers the advantage of reducing drug dosage and frequency, thereby minimizing adverse effects on patients.^[^ ^5^ ^]^ Upon the foundation of TPD, Proteolysis Targeting Chimera (PROTAC) technology stands out as a compelling and novel therapeutic modality.^[^ ^3^, ^5^ ^]^ As heterobifunctional ligands, PROTACs facilitate the interaction between POIs and E3 ligases, forming ternary complexes that exploit the ubiquitin‐proteasome system (UPS) for selective degradation of disease‐associated proteins^[^ ^6^ ^]^ as illustrated in **Figure** **1**. This strategy offers a more comprehensive approach compared to traditional small‐molecule inhibitors or receptor antagonists, effectively eliminating pathological protein function.^[^ ^3^ ^]^

Traditional research methodologies for PROTACs primarily rely on wet experimental approaches encompassing molecular design, synthesis, biological activity assessments, and clinical trials.^[^ ^1^, ^2^, ^7^ ^]^ While effective, these methods are often time‐consuming, labor‐intensive, and costly.^[^ ^3^, ^8^ ^]^ Recently, machine learning (ML)‐based approaches have rapidly progressed in PROTAC research.^[^ ^9^, ^10^ ^]^ Classic ML methods typically focus on specific data samples, constructing hybrid descriptors from PROTAC molecules and proteins, and harnessing algorithms to predict or optimize PROTAC properties like degradation capacity.^[^ ^11^, ^12^, ^13^, ^14^ ^]^ Deep learning (DL) methods, benefiting from more complex neural network designs, are capable of deriving latent representations of PROTAC molecules and proteins and capturing their intricate relationships.^[^ ^15^, ^16^ ^]^ These approaches encode PROTAC molecules and proteins as inputs, feeding them into deep neural networks such as Recurrent Neural Networks (RNNs).^[^ ^17^, ^18^, ^19^ ^]^ Graph Neural Networks (GNNs),^[^ ^18^, ^20^, ^21^, ^22^ ^]^ or Transformer architectures,^[^ ^22^, ^23^, ^24^, ^25^ ^]^ ultimately inferring target outcomes such as generated PROTAC molecules, degradation predictions, facilitating an end‐to‐end process.

Despite these significant advancements, current DL methods for PROTAC degradation prediction still face notable challenges. One key challenge is effectively modeling data from POIs, PROTAC molecules, and E3 ligases. Structural information plays a crucial role in interactions within the PROTAC system involving POIs, PROTAC molecules, and E3 ligases as the interactions are particularly related to substructures in PROTAC molecules and binding sites in the POIs and E3 ligases^[^ ^4^, ^26^ ^]^. PROTACs, as small molecules, inherently possess multi‐faceted characteristics, including molecular and atomic properties.^[^ ^27^ ^]^ However, they are frequently undercharacterized in research, with studies limited to SMILES representations or molecular fingerprints, failing to exploit the multi‐scale hierarchical information in PROTAC molecules.^[^ ^14^, ^18^, ^23^, ^24^ ^]^ Regarding POIs and E3 ligases, many existing methods overlook crucial protein structural information during data modeling. They often rely on raw protein descriptors like amino acid sequences to represent POIs and E3 ligases^[^ ^14^ ^]^ or sometimes omit the protein component entirely,^[^ ^17^, ^23^, ^24^, ^25^ ^]^ which tends to limit the model performance.

Another major challenge involves explicit learning of substructure interactions within the PROTAC system and the interpretability of computational outcomes. PROTAC molecules exert their effect by forming ternary complexes with POIs and E3 ligases, primarily through mutual substructure interactions among PROTAC molecules, POIs, and E3 ligases.^[^ ^1^, ^2^ ^]^ However, many previous studies have approached this by learning joint features in separate encoders, without explicitly capturing substructure interactions. In these methods, PROTAC, POI, and E3 ligase representations are initially learned separately, with mutual information being implicitly inferred through combination or a black‐box module.^[^ ^14^, ^18^, ^21^, ^22^ ^]^ which limits the depth of analysis in PROTAC system interactions. Moreover, the black‐box nature of these methods inherently restricts the interpretability of computational outcomes.^[^ ^28^ ^]^ Without explicit learning of substructure interactions, results become even more challenging to interpret, making it difficult for researchers to comprehend the biological basis underlying the computations.

To address these challenges, we propose PROTAC‐STAN, a structure‐informed deep ternary attention framework for interpretable PROTAC degradation prediction. PROTAC‐STAN integrates the hierarchical representation of PROTAC molecules, the structural embedding of POIs and E3 ligases, and a ternary attention network to model intermolecular interactions. For the first challenge, we encode hierarchical information in PROTAC molecules with molecular graph, SMILES word frequency mapping, and physicochemical properties across atom, molecule, and property hierarchies, respectively, via a custom Graph Convolutional Network (GCN),^[^ ^29^ ^]^ retaining critical features. For POIs and E3 ligases, we introduce crucial structural information into protein embedding for protein sequence data without requiring explicit protein structures as input inspired by ESM‐S,^[^ ^30^, ^31^ ^]^ capturing the protein structure information. For the second challenge, we introduce a novel ternary attention network tailored for the PROTAC system. Encoded representations are fed into the ternary attention network to learn substructure interactions among three entities. In this way, we can model interactions among three entities at the atom and amino acid levels, and utilize the learned ternary attention map to visualize the contribution of each substructure to the final result, aiding in revealing their binding mechanisms, thereby enhancing model interpretability.

Experiments on the enhanced public PROTAC‐DB^[^ ^32^ ^]^ with refined degradation information demonstrate that PROTAC‐STAN outperforms state‐of‐the‐art baselines in overall performance, substantially improving degradation prediction accuracy to 88.41%, with an AUROC of 0.8833 and an F1 score of 0.8588—an increase of more than 10% compared to the best baseline—while also enabling significant model interpretability through atomic‐ and residue‐level visualization of the molecule and complex. Our exploratory testing and case study through performance testing in specific scenarios, and MD simulation validation assessed the model's strong capability for real‐world applications. This computational simulation of the PROTAC system advances PROTAC research, paving the way for future therapeutic development. Our main contributions can be summarized as follows:

- We propose PROTAC‐STAN, a structure‐informed deep ternary attention framework for interpretable PROTAC degradation prediction integrating hierarchical representation of PROTAC molecules, structural embedding of POIs and E3 ligases, and ternary attention network modeling substructure interactions. Our approach effectively models data hierarchically and structurally, retaining critical features, and capturing structural information.
- We introduce a novel ternary attention network tailored for the PROTAC system, simulating substructure interactions among POIs, PROTAC molecules, and E3 ligases in nature and yielding interpretable outcomes, offering intuitive insights into PROTAC‐mediated protein degradation.
- Experiments on the enhanced PROTAC‐DB with refined degradation information show that PROTAC‐STAN outperforms state‐of‐the‐art baselines, improving overall performance while enhancing interpretability.

## 2 Results

### 2.1 PROTAC‐STAN Framework

**Figure 2:**
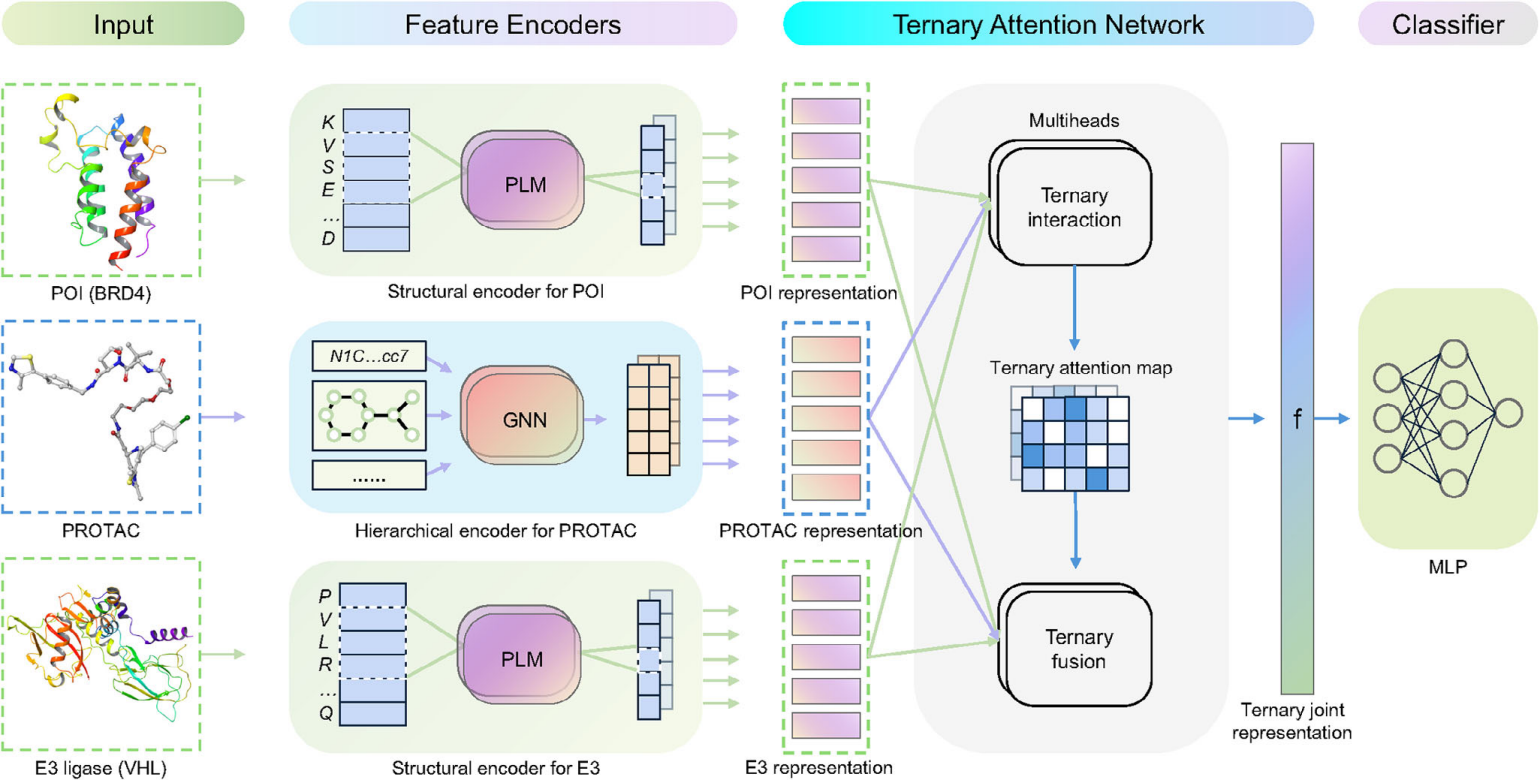
Overview of the PROTAC‐STAN framework. The input PROTAC molecule, POI (BRD4 example), and E3 ligase (VHL example) are first transformed into hierarchical PROTAC representation and structural POI/E3 representations via feature encoders. The PROTAC encoder encodes hierarchical information across atom, molecule, and property levels, while the structural encoder for POI and E3 ligase incorporates structural information into protein embeddings without needing explicit protein structures. The encoded representations then feed into the ternary attention network to learn substructure interactions. In the first ternary interaction step, the substructure representations of the three entities undergo ternary interaction modeling to generate a ternary attention map that captures interaction intensity. In the second ternary fusion step, these representations are fused into a joint POI‐PROTAC‐E3 representation **f** over the ternary attention map. Finally, an MLP classifier maps the joint representation to a degradation prediction result.

The PROTAC‐STAN framework, depicted in **Figure** **2**, consists of three feature encoder, a ternary attention network, and an MLP classifier. Given a (POI, PROTAC, E3 ligase) triplet, the inputs are first transformed by separate feature encoders: hierarchical encoder for PROTAC, and structural encoders for POI and E3 ligase. The hierarchical encoder, as detailed in Section 4, encodes hierarchical information for PROTAC, and the structural encoder integrates structural information for POI and E3 ligase, as explained in Section 4. Next, the encoded representations are fed into the ternary attention network to learn substructure interactions among the three entities, as described in Section 4. This network produces a joint POI‐PROTAC‐E3 representation, with a ternary attention map that visualizes the contribution of each substructure, enhancing model interpretability. Lastly, an MLP classification layer predicts degradation outcomes from the joint representation. By leveraging this framework, our model offers nuanced insights into PROTAC‐mediated protein degradation interactions, yielding more accurate predictions and improved result interpretability.

### 2.2 Performance Evaluations

#### 2.2.1 Performance on PROTAC‐fine

We enrich degradation information to the PROTAC‐DB 2.0^[^ ^32^ ^]^ as described in Section 4, and construct a refined PROTAC dataset named PROTAC‐fine which has a total size of 1,503 data samples. We divide PROTAC‐fine into train/test splits using a random 8:2 split, accounting for data leakage by avoiding having the same PROTAC samples in both splits, namely train/test+SMILES‐based setting. Data samples where a PROTAC was in the training set are moved out of the test set, leading to 1,202 training samples and 207 samples in the hold‐out test set. In order to compare with DeepPROTACs and PROTAC‐Degradation‐Predictor, we intersected PROTAC‐fine with PROTAC‐databank^[^ ^38^ ^]^, resulting in the bank‐fine dataset with PDBs containing 1213 data entries, and conducted replication comparison experiments. We applied a train/val/test + similarity‐based setting with an 8:1:1 split, preventing data leakage by assessing PROTAC SMILES similarity via the Tanimoto coefficient^[^ ^39^ ^]^ and E3 ligase and POI similarity using the Needleman‐Wunsch algorithm^[^ ^40^ ^]^ with a 0.9 similarity threshold. We trained the PROTAC‐STAN at the configuration of Table S3 (Supporting Information). Here, we evaluated the proposed PROTAC‐STAN with three baselines under two settings: support vector machine (SVM)^[^ ^33^ ^]^, random forest (RF)^[^ ^36^ ^]^, DeepPROTACs^[^ ^18^ ^]^ using accuracy, AUROC, and F1 score as metrics.

The comparison results are demonstrated in **Table** **1** Train(/val)/test evaluation. Between the two ML baselines, SVM performs slightly better than RF, and Morgan^[^ ^35^ ^]^ fingerprints feature extraction proves marginally more effective than MACCS^[^ ^34^ ^]^ fingerprints, which is consistent with findings from DeepPROTACs. We can see that DL methods perform better overall, especially in AUROC and F1 score, confirming that DL approaches are more adept at modeling complex data connections and are not easily biased in a single metric. Between the three DL methods, PROTAC‐STAN consistently outperforms DeepPROTACs and PROTAC‐Degradation‐Predictor by over 12% and 16% in accuracy, respectively, with similarly substantial improvements in AUROC and F1 scores across all experimental settings. Our data enrichment strategy enhances the model's ability to perform a more accurate and fitting prediction task, and the design of PROTAC‐STAN effectively captures hierarchical and structural information from refined data. Furthermore, ternary attention modeling of substructure interactions contributes substantially to the improvements in results. Specifically, PROTAC‐STAN reaches 88.41% in accuracy, with the highest AUROC at 0.8833, and also exhibited the highest F1 score at 0.8588. This highlights the superior performance of PROTAC‐STAN compared to previous methods, while also demonstrating its effectiveness in predicting PROTAC degradation. Furthermore, we demonstrate the interpretability of our PROTAC‐STAN method with ternary attention visualization in Section 2.3.

**Table 1:** Evaluation results of PROTAC‐STAN and baselines on test set considering data leakage. train/test+SMILES‐based: train/test splits by PROTAC SMILES; train/val/test+similarity‐based: train/val/test splits by similarity of (PROTAC, E3 ligase, POI), **Best**, Second, *Ours*.

Dataset	Setting	Method	Fingerprints	Accuracy	AUROC	F1 score
Train(/val)/test evaluation						
PROTAC‐fine	train/test SMILES‐based	SVM^[^ ^33^ ^]^	MACCS^[^ ^34^ ^]^	79.40%	0.7798	0.7355
			Morgan^[^ ^35^ ^]^	80.40%	0.7941	0.7547
		RF^[^ ^36^ ^]^	MACCS^[^ ^34^ ^]^	76.88%	0.7431	0.6806
			Morgan^[^ ^35^ ^]^	79.90%	0.7666	0.7059
		PROTAC‐STAN	—	***88.41%***	***0.8833***	***0.8588***
	train/val/test similarity‐based	SVM^[^ ^33^ ^]^	MACCS^[^ ^34^ ^]^	75.60%	0.7540	0.7355
			Morgan^[^ ^35^ ^]^	80.36%	0.8017	0.7871
		RF^[^ ^36^ ^]^	MACCS^[^ ^34^ ^]^	75.00%	0.7449	0.7083
			Morgan^[^ ^35^ ^]^	73.81%	0.7290	0.6615
		PROTAC‐STAN	—	***87.72%***	***0.8809***	***0.8814***
bank‐fine	train/test SMILES‐based	DeepPROTACs^[^ ^18^ ^]^	—	72.96%	0.8373	0.7152
		PROTAC‐Degradation‐Predictor^[^ ^37^ ^]^	—	69.50%	0.8137	0.6446
		PROTAC‐STAN	—	***85.98***%	***0.8601***	***0.8296***
	train/val/test similarity‐based	DeepPROTACs^[^ ^18^ ^]^	—	71.74%	0.7894	0.6829
		PROTAC‐Degradation‐Predictor^[^ ^37^ ^]^	—	66.67%	0.7684	0.6452
		PROTAC‐STAN	—	***84.62***%	***0.8436***	***0.8235***
Inference evaluation						
PROTAC‐DB 3.0	train/test SMILES‐based	SVM^[^ ^33^ ^]^	MACCS^[^ ^34^ ^]^	68.67%	0.6711	0.5795
			Morgan^[^ ^35^ ^]^	72.50%	0.7307	0.6551
		RF^[^ ^36^ ^]^	MACCS^[^ ^34^ ^]^	73.10%	0.6740	0.5574
			Morgan^[^ ^35^ ^]^	73.95%	0.6598	0.5149
		PROTAC‐STAN	—	***75.42%***	***0.7411***	***0.6644***
	train/val/test similarity‐based	SVM^[^ ^33^ ^]^	MACCS^[^ ^34^ ^]^	66.84%	0.6541	0.5606
			Morgan^[^ ^35^ ^]^	70.05%	0.7089	0.6316
		RF^[^ ^36^ ^]^	MACCS^[^ ^34^ ^]^	73.66%	0.6691	0.5416
			Morgan^[^ ^35^ ^]^	72.44%	0.6369	0.4682
		PROTAC‐STAN	—	***76.05%***	***0.7625***	***0.6912***

#### 2.2.2 Performance on PROTAC‐DB 3.0

Recently, PROTAC‐DB 3.0^[^ ^41^ ^]^ was relea sed, showcasing a great increase in data volume compared to version 2.0. The total number of entries in the PROTAC table has reached 9,380. However, the entries containing both explicit *DC*_50_ and *D*_*max*_ values remain relatively limited, with only 909 entries. Using the same data processing and enrichment methods in Section 4 applied to PROTAC‐DB 2.0, we compiled a dataset of 3,182 available entries labeled with degradation labels. We filtered out data that appeared in PROTAC 2.0, resulting in a dataset of 1,653 entries. We considered three strategies to evaluate the performance of PROTAC‐STAN on PROTAC‐DB 3.0. The first is the full dataset from PROTAC‐DB 3.0 inference evaluation. We evaluated the full PROTAC‐DB 3.0 with previous trained models under two settings as in Section 2.2.1. The second involves randomly sampling a test set of the same size in PROTAC‐DB 2.0 from the filtered set and conducting an out‐of‐distribution (OOD) test. The third strategy is to split the filtered dataset into a fine‐tuning set and a test set, where fine‐tuning involves initializing the model with the pre‐trained weights from PROTAC‐fine and then continuing training and evaluation on the sampled PROTAC‐DB 3.0 dataset. Following train/test+SMILES‐based setting, we split the filtered entries into 1,322 for fine‐tuning and 331 for testing. Using the same hyperparameter configuration, we conducted experiments with our method, SVM, and RF. The input POI/E3 ligase data of DeepPROTACs is unavailable, so we have to exclude it.

**Figure 3:**
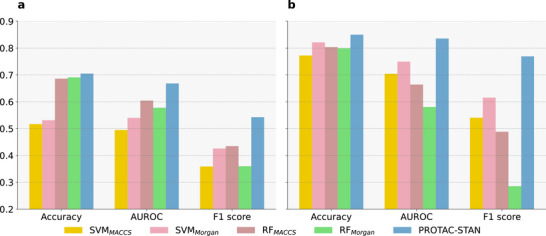
Evaluation on the filtered dataset constructed from the most recent PROTAC‐DB 3.0. Left (a) is out‐of‐distribution test, which is a inference evaluation directly on the sampled PROTAC‐DB 3.0 dataset. Right (b) is fine‐tune test, where fine‐tuning involves initializing the model with the pre‐trained weights from PROTAC‐fine and then continuing training and evaluation on the sampled PROTAC‐DB 3.0 dataset.

The full dataset evaluation results are shown in Table 1 Inference evaluation. PROTAC‐STAN outperforms all other baselines under two settings with accuracy at ≈75%. The highest AUROC and F1 score further validate its robustness. The results show that the model's performance is durable when presented with new data and performs well in predicting PROTAC degradation. The OOD and fine‐tune evaluation results are presented in **Figure** **3**. As shown, PROTAC‐STAN outperforms other methods in both tests, achieving the best overall performance. In the left of Figure 3, our model is the only one to exceed 70% accuracy and consistently leads in other metrics. This is remarkable because none of the test data samples were seen by our model beforehand, yet it performed relatively well on the OOD data, highlighting its robustness. The right of Figure 3 highlights that after fine‐tuning, our model gets significant improvements in accuracy, so did SVM and RF, suggesting that a more extensive PROTAC dataset could provide greater support for future PROTAC prediction tasks. We have observed that SVM improves a lot in accuracy, potentially due to its sensitivity to specific data features. SVMs and RF depend on particular features for categorization and face challenges with OOD data as shown in the left of Figure 3, after fine‐tuning on new data, their performance experiences improvement. However, their AUROC and F1 scores remained far lower than ours, confirming the resilience of our approach and greater flexibility and advantage of our model over other models in handling new data samples. Three experiments further underscore the overall capability of our method. Given the constraints imposed by limited training data in scale, there remains potential for enhancing our model's performance when applied to novel datasets. Notwithstanding these challenges, incorporating hierarchical and structurally encoded information alongside the utilization of ternary attention mechanisms has enabled our model to attain satisfactory results. This endeavor represents a promising exploration.

### 2.3 Interpretability With Ternary Attention Visualization

#### 2.3.1 Interpreting in 3D Attention Map

**Figure 4:**
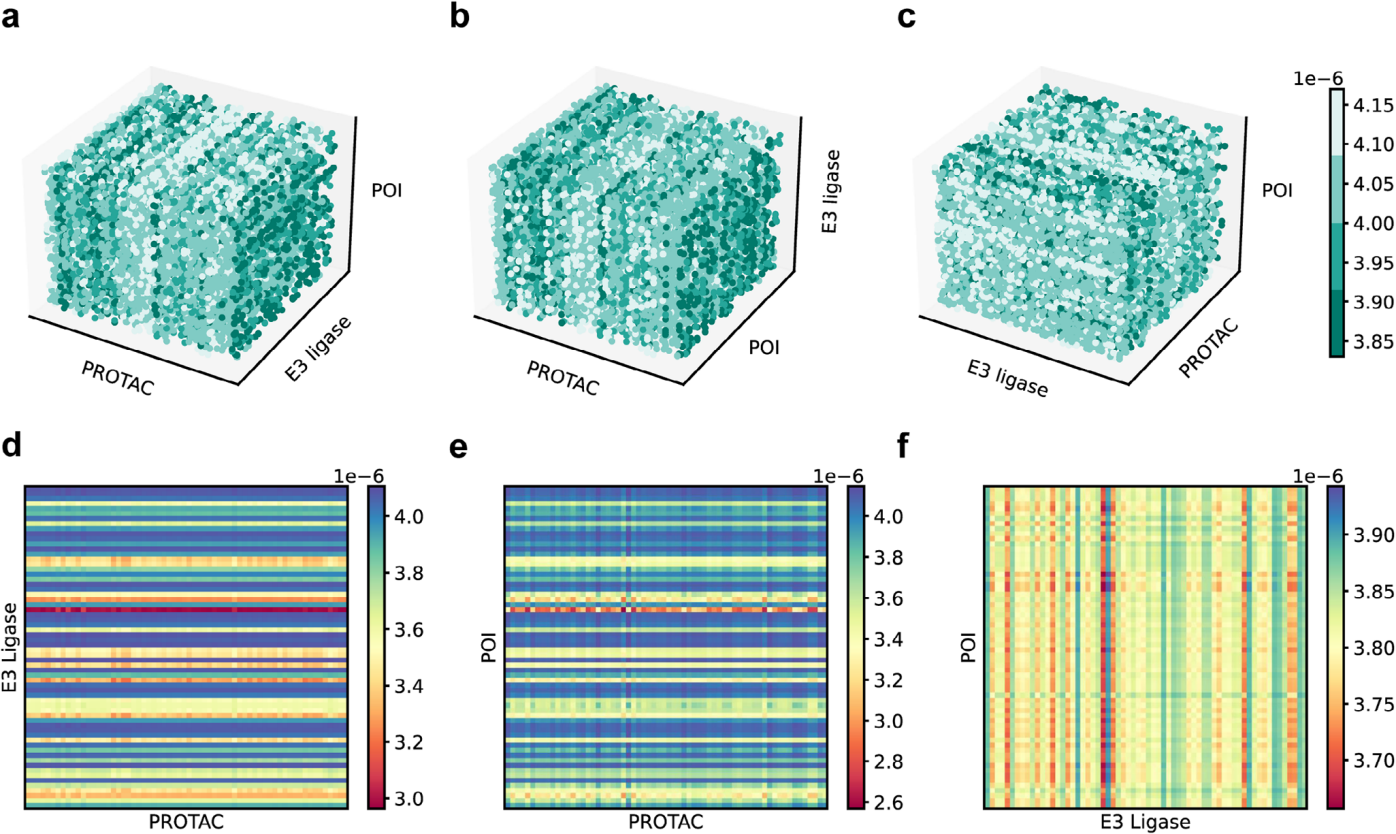
3D attention visualization in multi‐view and pairwise attention map visualization. a) Front view. Observation from front to back of attention map. Strong bar patterns of PROTAC interacting with POI. b) Top view. Observation from top to bottom of attention map. Strong bar patterns of PROTAC interacting with E3 ligase. c) Side view. Observation from left to right of attention map. Weak patterns between POI and E3 ligase. d) The PROTAC‐E3 ligase pairwise attention map. e) The PROTAC‐POI pairwise attention map. f) The E3 ligase‐POI pairwise attention map.

The strength of PROTAC‐STAN is to utilize the ternary attention map to provide interpretability and insights for understanding how each substructure in three entities contributes to the PROTAC degradation prediction results. We take one sample (Compound ID: 22) from PROTAC‐DB as an example and retrieve the generated three‐dimensional attention map after the ternary interaction process. We visually represent it in 3D across multiple perspectives, as shown in **Figure** **4**a–c. The shallow green hue signifies intense interaction, medium green denotes moderate interaction, and deep green indicates minimal or no interaction. In Figure 4a, the front view reveals discernible patterns in the PROTAC dimension's relationship to the POI dimension, suggesting specific interplays that significantly contribute to the formation of the ternary complex. In particular, we can observe that the feature images display vertical bars of varying shades. These distinct shades correspond to the interactions between specific locations on the PROTAC molecule and targeted regions on the POI during the learning process facilitated by ternary attention. Similarly, this pattern is evident in the top view in Figure 4b, further underscoring the importance of these interactions. Notably, the side view in Figure 4c exhibits weak patterns between POI and E3 ligase aligns with the detailed pairwise attention relationships presented in Section 2.3.2 indicating the comparatively weak interaction between POI and E3 ligase. Through direct 3D visualization of the ternary attention map, we can gain a macroscopic understanding of the potential interplay patterns among PROTAC, E3 ligase, and POI, which may facilitate our comprehension of their ternary complex formation process.

#### 2.3.2 Interpreting in 2D Attention Map

The 3D attention map quantifies the interactions among PROTAC, E3 ligase, and POI, allowing for the extraction of pairwise relationships in a straightforward manner. By employing a mean‐based approach along one dimension, we derive the other two pairwise attention maps, yielding the PROTAC‐E3 ligase attention map, the PROTAC‐POI attention map, and the E3 ligase‐POI attention map, as depicted in Figure 4d–f. The visualizations reveal that the attention values are notably elevated for PROTAC‐E3 ligase and PROTAC‐POI interactions, whereas relatively diminished for E3 ligase‐POI interactions. These intense values suggest the presence of specific regions in E3 ligase and POI, closely interacting with the PROTAC molecule. The medium E3 ligase‐POI interaction observed in Figure 4f corroborates findings in Section 2.6.2, which underscored the significance of protein‐protein interactions in PROTAC‐mediated degradation and their much weaker interaction compared to PROTAC‐E3 ligase/POI interactions. The 2D visualizations facilitate a deeper understanding of the pairwise relationships within the PROTAC system, providing valuable insights into PROTAC‐mediated protein degradation. More examples of attention map visualizations are provided in Figure S5– S8 (Supporting Information).

#### 2.3.3 Interpreting in Molecule and Complex

**Figure 5:**
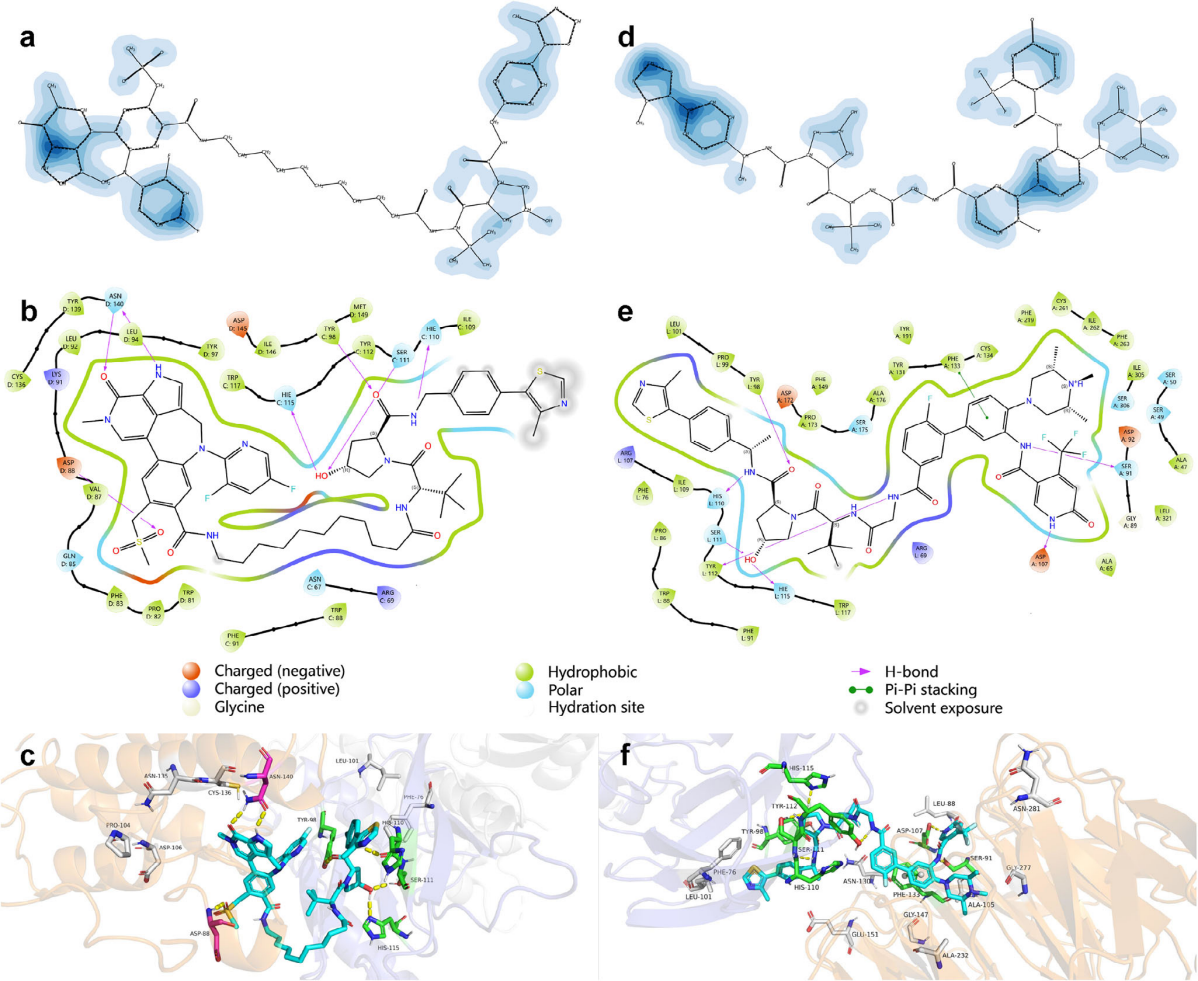
Ternary attention visualization on the PROTAC molecule and 3D Complex. Left (a–c) is from PDB 7KHH and right (d–f) from PDB 7JTP. a, d) 2D PROTAC molecule visualization with weighted atoms. The deeper the color, the higher the weight of the atom. b, e) 2D interaction visualization extracted from the complex with legend below.c,f) 3D pocket visualization with key residues. Top weighted residues are marked in gray, the actual interacting residues in green, and overlapped residues in magenta.

Beyond visualizing the attention maps, PROTAC‐STAN's capability can most importantly map these weights back onto the atom level of the PROTAC molecule and amino acid residue level of E3 ligase and POI, enabling interpretable analysis at molecular and complex levels. We mapped ternary attention back to the atomic level of PROTAC and to the residue level of E3 ligase and POI, and performed visualization on PROTAC molecules and 3D complexes. The visualized results are shown in **Figure** **5**, along with 2D interaction diagrams extracted from 3D complexes. Two examples (PDB ID: 7KHH^[^ ^42^ ^]^ and 7JTP^[^ ^43^ ^]^) were used to demonstrate this, where we obtained their 3D crystal structures from Protein Data Bank (PDB)^[^ ^44^ ^]^, prepared the proteins using Maestro, removed excess solvents, and filled in missing loops to obtain optimized structures.

We extracted the atomic weights of PROTAC from ternary attention maps and colored 60% of atoms, as shown in Figure 5a,d. The deeper the color, the higher the weight of the atom. We used Maestro to produce 2D interaction diagrams among PROTAC, E3 ligase, and POI from 3D complexes, with a range set at 3.5 Å, as shown in Figure 5b,e. We find the main interactions between PROTACs and proteins are hydrogen bonding and π‐π stacking interactions, with the interacting atoms mainly located at both ends of PROTAC, which is consistent with their actual binding modes (PROTAC warhead binds to POI, E3 ligand binds to E3 ligase). Take Figure 5a for example, the 2D molecular visualization of PROTAC accurately identified multiple key interaction regions, as well as corresponding atoms or groups. Specifically, in the left part of the molecule in Figure 5b, the oxygen atom (C = O) from the 2‐Pyridone ring interacts with residue ASN D:140 (D denotes Chain D), and the NH group from the adjacent pyrrole ring also interacts with ASN D:140, forming stable hydrogen bonds. The oxygen atom (S = O) from the methylsulfonyl group interacts with ASP D:88, forming a hydrogen bond. In the right side of the molecule in Figure 5b, the hydroxyl group (OH) from the 3‐hydroxypyrrole ring interacts with residue SER C:111 and HIE C:115 (C denotes Chain C), forming hydrogen bonds. Additionally, the carbonyl oxygen atom (C = O) from the amide group interacts with TYR C:98, and the nitrogen atom (NH) from the amine group interacts with HIE C:110, also forming hydrogen bonds. These visualizations highlight the key interaction regions where PROTAC interacts with E3 ligase and POI, suggesting that these areas of PROTAC may play a critical role in the formation and stability of the ternary complex.

Figure 5c,f show the binding situation within 3D pockets. We used PyMOL to implement this. From ternary attention maps, we extracted the top five weighted residues of E3 ligase and POI, marked the top important weights in gray, and marked the actual interacting residues in green, overlapped residues in magenta, hydrogen bonds in yellow, and π–π stacking interactions in green. The blue‐purple part in background represents E3 ligase, while the orange part represents POI. We computed the distances of these residues to the centroid of the PROTAC molecule to assess spatial distribution and approximate the interaction interface in Table S4 (Supporting Information). The results show that most top‐weighted residues are located near interaction residues, with few residues extending beyond these interaction sites. Among the labeled residues, the proportion of weighted residues in 7KHH reaches over half, and the correct trend perception in 7JTP is observed. In Figure 5c, it can be seen that residue ASN‐140 and ASP‐88 were accurately marked as high‐weight residues, which formed hydrogen bonding interactions with PROTAC molecules. Other top‐weight residues not forming interactions, such as CYS‐136, and PHE‐76, are also located near the interacting residues, which is quite impressive and indicates that our method can provide a certain level of interpretability for 3D complexes. In Figure 5f, the interacting residues are relatively concentrated, although there are no overlapping residues. However, our high‐weight residues such as PHE‐76, LEU‐88, ASN‐130, GLY‐147, and ALA‐105 are all very close to the interacting residues, which suggests that our ternary attention learning has a certain trend perception ability for key residues and can potentially help us understand PROTAC forming ternary complexes.

### 2.4 Exploratory Testing

#### 2.4.1 Linker Sensitivity

**Figure 6:**
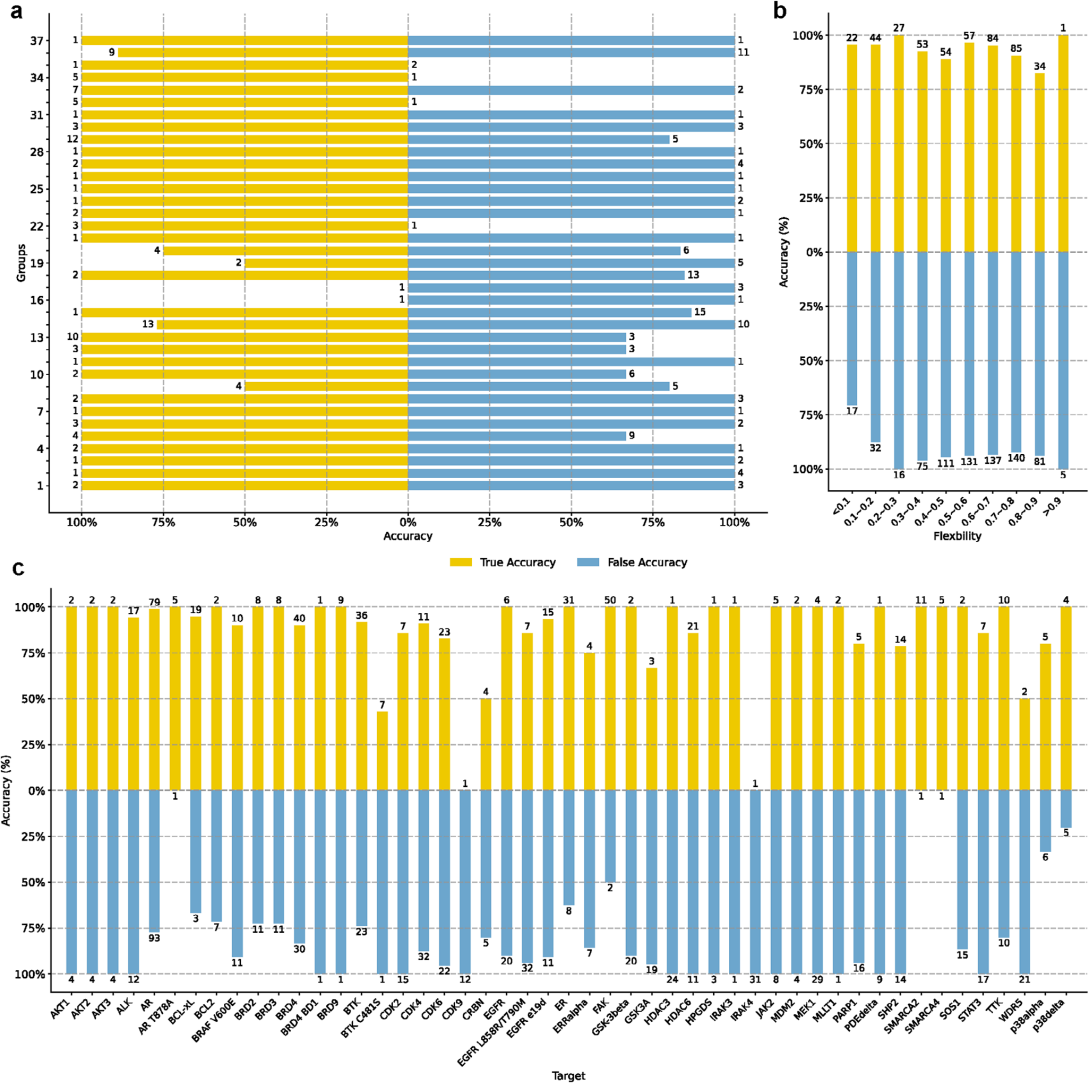
Exploratory evaluation results. a) Linker sensitivity evaluation through grouped degradation prediction. Samples in each group have fixed E3 ligase‐E3 ligand and POI‐warhead pair, and varied linker. b) Evaluations across different linker flexibility groups. Higher flexibility values indicate more flexible and lower values indicate more rigid. c) Per‐target evaluation. The number of both ends are sample numbers in each group.

The linker in PROTAC plays a pivotal role in determining its biodegradative efficacy^[^ ^45^ ^]^, serving as a critical component that influences many aspects of the design and function of PROTAC. We investigate the sensitivity of our method to linker variations. We aligned the PROTAC samples in our refined dataset with additional data table containing warheads, linkers, and E3 ligands^[^ ^32^ ^]^, resulting in a total of 1,323 data entries, which include (PROTAC, warhead, linker, E3 ligand) matches. We then grouped these entries by (warhead, E3 ligand) pairs and discarded groups containing only true or false samples, yielding 251 data entries across 37 groups. We evaluated all samples using PROTAC‐STAN, achieving a degradation prediction accuracy of 88.45%, showing our method's excellence. We then conducted experiments for each group, and the results are presented in **Figure** **6**a. Among the 37 groups, accurate predictions for both true and false samples were observed in 20 groups, with overall accuracy being relatively balanced between true and false predictions. We observed that groups exhibiting 0% prediction accuracy primarily consist of single samples, which highlights the urgent need for more PROTAC data to enhance our current method. In each group, the E3 ligase‐E3 ligand and POI‐warhead remained constant while the linker varied. Our method effectively predicted the correct degradation outcomes, indicating that PROTAC‐STAN exhibits sensitivity to linker variations during degradation predictions. This finding enhances the applicability of PROTAC‐STAN, enabling its future integration with linker design methodologies for the discovery of new PROTAC candidates.

#### 2.4.2 Linker Flexibility

Linker flexibility is a critical aspect of PROTAC design, significantly influencing model performance in real‐world applications. Current models predominantly focus on conventional linkers, such as PEG or alkane chains^[^ ^18^ ^]^, whereas clinically relevant rigid linkers require further evaluation. To assess model performance on PROTACs with varying linker flexibility, we calculated linker flexibility scores following the method described in Ref. [46]. This score is defined as the ratio of rotatable bonds to the total number of bonds, where higher values indicate greater flexibility and lower values indicate increased rigidity. We then categorized linkers based on their flexibility and evaluated the model's performance within each group as in Figure 6b. PROTAC‐STAN exhibited marginally reduced stability when processing rigid linkers compared to its performance with more flexible linkers, while still maintaining competitive effectiveness. This observed performance variation correlates with the corresponding training dataset sizes, suggesting that data imbalance across linker categories likely contributes to these fluctuations. Moreover, prospective validation (Section S2.5, and Section S1.1.3, Supporting Information) using PROTACs with uncommon linkers confirmed robust accuracy for moderately flexible linkers (PROTAC 1, 4, 12, 13) but a slight drop for highly rigid ones (PROTAC 10, 11), though still within competitive thresholds. These findings underscore the need for dataset expansion to improve generalizability across diverse linker chemotypes.

#### 2.4.3 Target Adaptation

To further assess the model's robustness and generalizability, we performed a per‐target analysis, which is now presented in Figure 6c. We grouped the data based on the specific targets of PROTACs and evaluated the model's predictive performance on a per‐target basis. Our experimental results demonstrate that PROTAC‐STAN consistently delivers stable predictive performance across major targets, with its outcomes showing strong alignment with comprehensive evaluation metrics. While a marginal performance decline is observed for targets with limited training data ‐ a predictable outcome given the inherent data constraints ‐ the model maintains competitive performance levels. These findings underscore the significance of dataset expansion in further improving model robustness and generalizability across diverse target scenarios. Furthermore, our fine‐tuning experiments in Figure 3 using new data, observed a significant improvement in predictive performance. This suggests that the model can effectively integrate a few new data samples and adapt to novel targets beyond the training set, which is crucial for real‐world applications.

### 2.5 Case Study

To demonstrate the predictive capability of PROTAC‐STAN for novel PROTACs, we selected cyclin‐dependent kinase 4 (CDK4) as a case study. CDK4 (UniProt ID: P11802) is a Ser/Thr kinase and a critical regulator of the cell cycle. It serves as a therapeutic target for various malignancies, including breast cancer, malignant melanoma, and non‐small cell lung cancer. PROTAC‐based degraders targeting CDK4 offer a promising strategy for cancer treatment, potentially overcoming acquired resistance associated with traditional CDK4/6 inhibitors such as palbociclib. In this study, we utilized palbociclib (a known CDK4 inhibitor) as the ligand targeting CDK4 and a known von Hippel‐Lindau (VHL) binder (PubChem Compound CID: 129900323) as the E3 ligase ligand. 13 linkers with distinct lengths and chemical structures were designed (Table S2, Supporting Information). After constructing ternary complexes (CDK4‐PROTAC‐VHL) for all 13 PROTACs (Data S1, Supporting Information), 500 ns molecular dynamics (MD) simulations were performed to evaluate structural stability.

#### 2.5.1 Binding Free Energy Analysis

**Figure 7:**
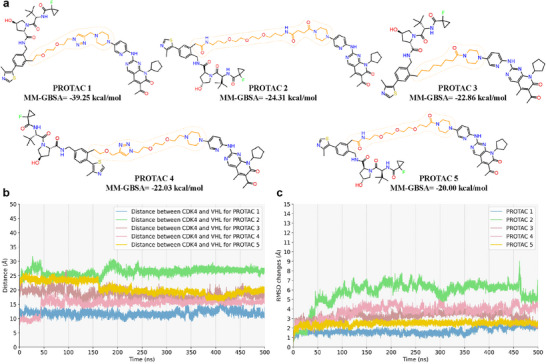
MD simulation results. a) Top five PROTACs with MM‐GBSA results. The top five PROTACs, which were predicted to have degradation activity by the PROTAC‐STAN model, exhibited enhanced binding affinity between the CDK4 protein and the VHL protein, as determined by MM‐GBSA calculations from MD simulations. b) Distances between CDK4 and VHL over time. The smaller the distance changes, the more stable the complex. c) RMSD changes of PROTAC over time. The smaller the RMSD value, the more stable the binding of PROTAC to the proteins.

The binding free energy calculated by MM‐GBSA methods revealed strong protein‐protein interactions (ranging from ‐39.25 to ‐20 kcalmol^−1^) for PROTAC 1‐5, aligning with their high degradation activity predicted by PROTAC‐STAN (**Figure** **7**a). PROTAC 6 and 7 showed moderate binding affinity (‐15 to ‐20 kcalmol^−1^), consistent with model predictions. In contrast, PROTAC 8‐13 induced weak protein‐protein interactions (MM‐GBSA > ‐15 kcalmol^−1^). Notably, PROTAC 12 and 13 exhibited system collapse during the initial simulation phase, likely due to unstable ternary complexes. Among these six PROTACs with low ternary complex stability, four (PROTAC 8, 11, 12, and 13) were classified as low‐activity degraders by PROTAC‐STAN (Table S2, Supporting Information). These results highlight PROTAC‐STAN's ability to prioritize candidates with high ternary complex stability while filtering out low activity degraders. As a false‐positive case, PROTAC 9 was incorrectly predicted as high‐activity, but upon closer inspection, we found that it is structurally almost identical to PROTAC 1, differing by only one PEG unit in the linker. Their high structural similarity led to close prediction scores, which demonstrate that mispredictions often stem from high structural similarity with known degraders, rather than from fundamental flaws in the model. This further supports the model's robustness. Correlation analysis between PROTAC‐STAN's prediction score and the MM‐GBSA (Section S1.1.4, Supporting Information) further supports the model's practical reliability.

#### 2.5.2 Protein‐Protein Distance Analysis

We assessed the spatial distance between CDK4 (residue Arg101) and VHL (residue Tyr98) over time by measuring their separation. The top five results are shown in Figure 7b. Overall, the distance between CDK4 and VHL remained relatively stable across all five samples, indicating that the ternary complex maintained its structural integrity during the molecular dynamics simulation, with no significant conformational changes. This result suggests that the PROTAC molecule successfully induced the formation of a stable ternary complex between CDK4 and VHL, further supporting the experimental conclusion that PROTAC 1‐5 exhibits high degradation activity. It also validates the accuracy and effectiveness of our deep learning model in predicting PROTAC degradation activity.

#### 2.5.3 Root Mean Square Deviation (RMSD) Analysis

We conducted RMSD analysis on the conformational stability of the ternary complex based on PROTAC molecules to evaluate its structural changes during molecular dynamics simulations. The top five results are shown in Figure 7c. From the RMSD trends, PROTAC 1 and PROTAC 3‐5 exhibited high conformational stability throughout the simulation, while PROTAC 2 showed slight fluctuations at certain time points. However, the overall RMSD changes were minimal, indicating that the ternary complex remained stable. The maximum RMSD for PROTAC 2, which exhibited the largest fluctuations, was approximately 8 Å, while the maximum RMSD for the other PROTAC molecules was around 5 Å, demonstrating good binding stability of the PROTAC ligands in the target protein binding pocket. These results further confirm the high stability of the ternary complex induced by PROTAC 1‐5, consistent with the predictions of our deep learning model.

#### 2.5.4 Solvent‐Accessible Surface Area (SASA) Analysis

We evaluated the degradation potential of PROTAC‐induced ternary complexes by analyzing the SASA of lysine residues. The top five results are shown in **Table** **2**. The analysis revealed that the ternary complexes induced by PROTAC 1‐5 exhibited large SASA values, with an average of approximately 70 Å^2^ and a maximum of up to 136 Å^2^, indicating high solvent exposure during the simulation. Higher SASA values are generally associated with increased recognition and ubiquitination by E3 ligases^[^ ^47^ ^]^, suggesting that PROTAC 1‐5 likely possess high degradation activity. This conclusion aligns closely with the predictions of our deep learning model, further validating its effectiveness in predicting PROTAC degradation activity.

**Table 2:** SASA of lysine statistics during simulation.

Complex	Max area (Å^2^)	Min area (Å^2^)	Average area (Å^2^)
CDK4‐PROTAC 1‐VHL	136.17	17.11	74.2
CDK4‐PROTAC 2‐VHL	125.67	6.35	65.54
CDK4‐PROTAC 3‐VHL	128.07	13.28	72.31
CDK4‐PROTAC 4‐VHL	130.43	−2.76	75.01
CDK4‐PROTAC 5‐VHL	131.64	4.22	68.72

### 2.6 Ablation Study

#### 2.6.1 Ablating Components of PROTAC‐STAN

Here, we perform ablation experiments to evaluate the key components of the PROTAC‐STAN framework introduced in Section 2.1. To this end, we use a series of ablation schemes. The Raw scheme replaces each component in PROTAC‐STAN with the PROTAC SMILESNet encoder,^[^ ^18^ ^]^ the POI/E3 ligase Ngrams encoder,^[^ ^14^ ^]^ and concatenation fusion^[^ ^14^, ^18^ ^]^ for comparison. We then analyze the impact of each component individually. Specifically, the ‐S1 scheme evaluates the effect of the PROTAC hierarchical encoder, the ‐S2 scheme evaluates the effect of the POI/E3 ligase structural encoder, and the ‐S scheme combines ‐S1 and ‐S2 to assess the overall effect of feature encoding. Additionally, the ‐T scheme evaluates the influence of the ternary attention network. Lastly, we compare the overall performance enhancement of PROTAC‐STAN against the Raw scheme. From **Table** **3**, it is evident that the Raw model constructed from baselines performs adequately in predicting degradation, thus validating previous work. The ‐S model improves the F1 score from 0.7738 to 0.8333, highlighting the effectiveness of our structure‐informed feature encoders, with ‐S1 more contributing than ‐S2. The ‐T model is comparable to Raw due to the simple encoding of Raw; however, the notable difference lies in that ‐T introduces a ternary attention network to fuse POI, PROTAC, and E3 ligase as well as modeling ternary interactions among them. Moreover, incorporating ‐S and ‐T components, i.e., PROTAC‐STAN, results in a 6.77% improvement in accuracy and achieves 0.8833 in AUROC and 0.8588 in F1 score, thereby validating the superior performance of our method. Regarding the hierarchical encoder's SMILES maximum length and the structural encoder's PLM, we conducted additional ablation tests in Section S1.3.2 (Supporting Information). By comparing models with a length of 256 or PLM as ESM‐2, we observed performance degradation in both settings. This confirms that the 128‐length configuration is better suited for the current task, while ESM‐S, with its explicit structural supervision, delivers additional benefits in this specific scenario.

**Table 3:** Ablation Study on PROTAC‐STAN Components. The Raw scheme is a base constructed from baselines, the ‐S1 and ‐S2 schemes ablate the PROTAC hierarchical and POI/E3 ligase structural encoders. The ‐S scheme combines ‐S1 and ‐S2 to assess feature encoding effects. The ‐T scheme evaluates the ternary attention network over concatenation. **Best**, Second.

Component	Raw	−S1	−S2	−S	−T	−STAN
SMILESNet encoder^[^ ^18^ ^]^	✓		✓		✓	
Hierarchical encoder		✓		✓		✓
Ngrams encoder^[^ ^14^ ^]^	✓	✓			✓	
Structural encoder			✓	✓		✓
Concatenation^[^ ^14^, ^18^ ^]^	✓	✓	✓	✓		
TAN fusion					✓	✓
Accuracy	81.64%	85.99%	81.64%	85.51%	80.68%	**88.41%**
AUROC	0.8109	0.8671	0.8229	0.8631	0.8108	**0.8833**
F1 score	0.7738	0.8380	0.7889	0.8333	0.7753	**0.8588**

#### 2.6.2 Ablating Ternary Attention Network (TAN)

We further investigate the TAN module of PROTAC‐STAN by examining different fusion methods for POI, PROTAC, and E3 ligase features, varying TAN heads, and input orders. We compare TAN fusion against concatenation fusion^[^ ^14^, ^18^ ^]^ and LMF^[^ ^48^ ^]^, a low‐rank multimodal fusion method originally designed for visual, audio, and language inputs. As shown in Table S11 (Supporting Information), Concatenation and LMF are comparable to TAN_1_ but perform less well than TAN_2_. This indicates that all three methods are capable of fusing the three features, but in terms of methods, Concatenation is significantly naive compared to LMF and TAN. LMF and TAN are more complex to train, which means they are better performed at the same complexity. Additionally, TAN introduces a ternary attention map, providing better interpretability compared to Concatenation and LMF. For multi‐head configurations, we find that multiple TAN heads outperform a single head, but with increased parameters. This is why we opted not to add more heads, considering the parameter growth and dataset volume. Additionally, we analyze the impact of different input permutations for POI, PROTAC, and E3 ligase, revealing that the order of inputs has a minimal effect on final performance. The permutations 123 and 312 (corresponding to PROTAC, E3 ligase, and POI) yield the best results, while other orders show similar performance, indicating that the influence of input feature order is minor.

Notably, PROTAC‐STAN_*pair*_ is a variant that focuses on pairwise interaction, i.e. the PROTAC and POI pair, the PROTAC and E3 ligase pair, rather than considering all three entities simultaneously of PROTAC‐STAN. The performance of this variant was worse than that of PROTAC‐STAN, suggesting that the interaction between proteins may play a crucial role in PROTAC‐mediated degradation, a factor that existing methods had not accounted for. This is a fascinating finding that also aligns with and supports recent research^[^ ^49^, ^50^, ^51^ ^]^.

### 2.7 Model Efficiency

We conducted a computational efficiency evaluation in this section, measuring inference time and memory consumption between DeepPROTACs and our model. For a fair comparison, we conducted tests on the same machine using the bank‐fine dataset, setting the batch size to four for all models. The results, now presented in **Table** **4**, show that PROTAC‐STAN has a training speed 1.5x faster than DeePROTACs and uses 13% less memory in training and 12% less in inference. This efficiency is attributed to the parameter sharing mechanism in our TAN network architecture, coupled with the optimized einsum computation, which collectively demonstrate the superior performance of our method. While TAN incurs additional computational costs in GPU memory, these are within an acceptable range. More importantly, the improved interaction modeling and enhanced interpretability provided by TAN justify this trade‐off. As part of our future work, we aim to explore potential optimizations, such as sparsity‐based attention mechanisms or model distillation, to further improve efficiency while maintaining predictive power.

**Table 4:** Computational efficiency comparison between DeepPROTACs and PROTAC‐STAN.

Mode	Metrics	DeepPROTACs	PROTAC‐STAN	PROTAC‐STAN (save attention map)
Train	Time/epoch	9.23 seconds	5.9 seconds	—
	System memory	3374 MB	2918 MB	—
	GPU memory	1273 MB	3465 MB	—
Inference	Time/sample	0.568 seconds	0.508 seconds	0.536 seconds
	System memory	3165 MB	2812 MB	2821 MB
	GPU memory	1199 MB	913 MB	1269 MB

## 3 Discussion

In this study, we introduce PROTAC‐STAN, an end‐to‐end structure‐informed deep ternary attention framework designed for interpretable PROTAC degradation prediction. Our approach integrates hierarchical representations of PROTAC molecules, structural embeddings of POIs and E3 ligases, and a ternary attention network to model substructure interactions effectively. By leveraging hierarchical and structural data modeling, PROTAC‐STAN retains essential features and captures critical structural information. The novel ternary attention network, specifically tailored for the PROTAC system, simulates substructure interactions among POIs, PROTAC molecules, and E3 ligases, yielding interpretable outcomes and providing intuitive insights into PROTAC‐mediated protein degradation.

Experiments across diverse datasets demonstrate that PROTAC‐STAN outperforms state‐of‐the‐art deep learning models and traditional machine learning approaches in predicting PROTAC degradation. Beyond overall performance, our approach differs fundamentally from recent predictors such as DeepPROTACs^[^ ^18^ ^]^ and PROTAC‐Degradation‐Predictor^[^ ^37^ ^]^ in input representation, interaction‐modeling paradigm, and the level of interpretability. DeepPROTACs decomposes a PROTAC into warhead, linker, and E3‐ligand branches; the POI and E3 binding pockets are represented as graphs, and all branches are concatenated before the classification head. This design captures pocket‐level geometric/topological cues but relies on a late‐fusion strategy in which modalities are encoded separately and then concatenated. PROTAC‐Degradation‐Predictor represents the PROTAC with Morgan fingerprints, combines pretrained protein embeddings, and incorporates cell‐line context via text embeddings; modality outputs are linearly projected, weighted‐summed, and fed to a shallow prediction head. While PROTAC‐Degradation‐Predictor can leverage external cellular context, its cross‐modal fusion remains comparatively coarse‐grained. In contrast, PROTAC‐STAN uses a hierarchical multi‐scale PROTAC encoder that aggregates features at the atom, fragment, and molecule levels. Combined with structure‐informed protein representations, this enables explicit modeling of PROTAC–POI–E3 interactions via a ternary attention network. This design yields atom and residue‐level attributions and ternary attention maps that connect global interaction patterns with fine‐grained mechanistic hypotheses. Consistently, on intersected comparable subsets, PROTAC‐STAN achieves superior performance, and exhibits stronger OOD robustness and few‐shot adaptability. In terms of use cases, PROTAC‐Degradation‐Predictor can be attractive when rich cell‐line annotations dominate the signal, whereas PROTAC‐STAN is preferable for structure‐guided design and mechanistic interpretation and for cross‐target generalization in early discovery. We further corroborated PROTAC‐STAN's practical utility through targeted case studies and MD‐based analyses, which align the model's attention‐derived hypotheses with plausible interaction motifs, thereby informing linker optimization, POI/E3 selection, and hypothesis‐driven experimental design.

Our exploratory testing and case study through predictive performance testing in specific scenarios, and MD simulation validation assessed the model's strong capability for real‐world applications. The architecture of PROTAC‐STAN facilitates an in‐depth understanding of how PROTACs interact with proteins and ligases, moving beyond traditional models that only consider binary interactions. This comprehensive approach enables the model to capture complex interaction dynamics, contributing to the advancement of PROTAC drug discovery and offering insights into optimizing drug design for enhancing efficacy and selectivity.

Notably, the analysis of E3‐POI interactions highlights the critical role of system cooperativity in achieving effective target protein degradation. Previous studies have shown that such interactions are often inherently weak,^[^ ^52^ ^]^ with factors such as linker length playing a crucial role in the formation and stability of the ternary complex.^[^ ^53^ ^]^ For instance, shorter linkers have been associated with more efficient target protein degradation. The stability and efficiency of the ternary complex are strongly dependent on the cooperativity of the system, suggesting that even weak E3‐POI interactions can be optimized through rational PROTAC design. These findings emphasize the broader potential to utilize weak interactions by enhancing system cooperativity, supporting the idea that careful molecular design can overcome the limitations of weak binding affinities and achieve biologically effective degradation.

In addition, this study has certain limitations, including dataset bias and the lack of detailed protein structural information. Regarding dataset bias–particularly the lower performance on PROTACs with rigid linkers due to their underrepresentation in the training data–we plan to address this issue by prioritizing the collection of such data in future studies to enhance the scope and generalizability of the model. Looking ahead, as PROTAC data continue to accumulate, we will systematically harvest additional structures and activity annotations from the primary literature and patent databases, with particular emphasis on rigid and macrocyclic linkers, to correct dataset imbalance and scarcity. For the lack of detailed protein structural information, this study attempts to mitigate the impact by employing ESM‐S, a protein representation encoder pre‐trained on large‐scale datasets containing both sequence and structural information. ESM‐S is capable of capturing latent structural features related to binding pocket characterization solely from sequences, thereby partially alleviating the limitations caused by the absence of experimental structural data. However, ESM‐S may still introduce biases when encoding binding pocket residues, particularly when dealing with post‐translational modifications (PTMs) or dynamic conformational changes. To overcome this limitation, we plan to incorporate predicted structures from the AlphaFold3 series in future research to further improve prediction accuracy and better account for structural variability.

In conclusion, PROTAC‐STAN has already achieved notable success in surpassing other similar algorithms. We anticipate that with the future expansion of PROTAC datasets, this model will demonstrate enhanced robustness. Furthermore, incorporating a broader range of information is expected to further improve the model's overall performance.

## 4 Experimental Section

### Data Preparation

*Dataset* The original data from PROTAC‐DB 2.0^[^ ^32^ ^]^ was sourced, which was structured into four main tables detailing information on PROTACs, Warheads, Linkers, and E3 Ligands. The database includes chemical structures, biological activities, and physicochemical properties extracted from experimental literature. The primary table for PROTACs comprises 5388 entries, representing 3270 unique PROTAC molecules. Duplicate entries typically denote the same molecule tested under different experimental conditions. Each entry included the PROTAC's SMILES, the POI and E3 ligase's UniProt ID. Crucially, the dataset contains information on PROTAC degradation activity, including *DC*_50_ (half‐maximal degradation concentration) and *D*_*max*_ (maximum level of protein degradation). These metrics were standard indicators of a PROTAC's capability to degrade its target protein^[^ ^54^ ^]^, with lower *DC*_50_ and higher *D*_*max*_ values suggesting higher degradation activity.

*Data Enrichment*

Due to a significant proportion of PROTACs in the original database lacking *DC*_50_ and *D*_*max*_ data, the availability of degradation‐informed data was notably limited, posing challenges for deep learning applications. Among the 5,388 entries cataloged, merely 362 possess detailed records of both *DC*_50_ and *D*_*max*_ values. The in‐depth analysis of the database in Section S1.2.7 (Supporting Information) indicated that it was possible to derive additional degradation activity information from experimental descriptions related to *Assay* (*DC*_50_/*D*_*max*_ *)*, Percent degradation, and Assay (Percent Degradation). Specifically, for each entry, explicit *DC*_50_ or *D*_*max*_ values were first check; if none were present, implied *DC*_50_ and *D*_*max*_ values were first extracted from available experimental descriptions. This effort had enabled us to preliminarily yield 1631 entries, effectively increasing the original data volume by 350%.

*Degradation Labeling*

The quantification of a PROTAC's ability to degrade target protein was commonly assessed using *DC*_50_ and *D*_*max*_^[^ ^54^ ^]^. To effectively train the deep learning model, a robust labeling protocol based on that used in DeepPROTACs^[^ ^18^ ^]^ was established for processing degradation labels. This protocol considered both explicit data points, where *DC*_50_ and *D*_*max*_ values were directly available, and implicit scenarios where these values need to be inferred from experimental observations. This tiered protocol allowed to effectively utilize available data and train the model for accurate PROTAC degradation prediction. The specific criteria are as follows:

Following these labeling criteria, 620 entries were identified as possessing high degradation activity and 1,011 entries as low. The refined dataset was named PROTAC‐fine and make it publicly available. These enhancements to PROTAC‐DB significantly bolster the database's comprehensiveness, offering substantial support for future research and applications in the field.

### PROTAC‐STAN Architecture

*Problem Formulation*

In PROTAC degradation prediction, the task was to determine whether a triplet of a POI, a PROTAC molecule, and an E3 ligase would form a ternary complex to degrade the POI via the UPS. Given a POI $P_{p}$, a PROTAC molecule G, and an E3 ligase $P_{e}$, PROTAC degradation prediction aims to learn a model M to map the joint feature representation space $P_{p}\times G\times P_{e}$ to a degradation label *l* ∈ {0, 1}, where 1 indicates high degradation and 0 indicates low degradation. Major notations used in this paper are provided in Table S1 (Supporting Information). The PROTAC‐STAN architecture was presented step by step, and the overview of PROTAC‐STAN is illustrated in Figure 2.

*Hierarchical Encoder for PROTAC Molecules*

**Figure 8:**
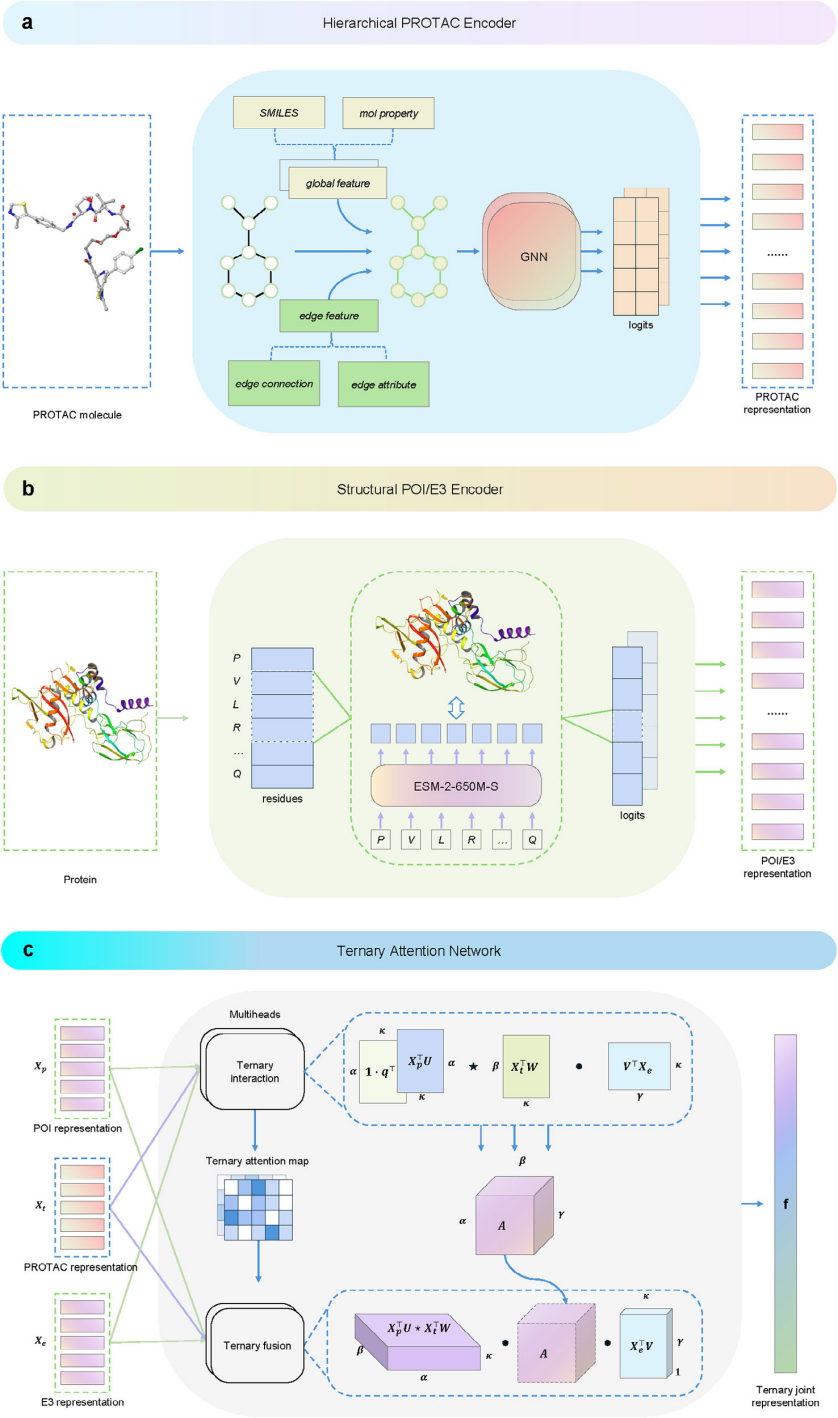
PROTAC‐STAN components. a) Hierarchical PROTAC encoder. Given a PROTAC molecule, the encoder represents it across atom, molecule, and property hierarchies. b) Structural protein encoder. Given a POI or E3 ligase, the encoder transforms its amino acid sequence into a structure‐informed representation. c) Ternary attention network. Given the POI, PROTAC and E3 representation **X**_*p*_, **X**_*t*_ and **X**_*e*_, a ternary attention map **A** and joint POI‐PROTAC‐E3 representation **f** are obtained. ⋆ denotes the Einstein summation convention *ik*, *jk* → *ijk*, and · denotes the matrix multiplication.

The hierarchical molecular feature encoder transforms an input PROTAC molecule into a hierarchical 2D molecular graph $G\left(\langle V,H\rangle ,E\right)$ as illustrated in **Figure** **8**a. For node information V, each atom node was initially defined by its chemical properties using the PyG package. Each atom was characterized by nine atomic features: atomic number, chirality, degree, formal charge, number of hydrogen atoms, number of radical electrons, hybridization state, aromaticity, and ring membership. Consequently, the node feature for each graph is denoted as $V=\{v_{1},v_{2},\text{…}\}$, where *v*_*i*_ is the *i*‐th node feature, with |V| represents the number of nodes. Regarding edge information E, it is represented as $E=(E_{e},E_{a})$, where $E_{e}\in R^{\varepsilon \times 2}$ denotes the edge connections, and $E_{a}\in R^{\varepsilon \times 3}$ encodes three bond attributes: bond type, bond stereochemistry, and conjugation status, with ϵ represents the number of edges in the graph.

To represent hierarchical information in G, a global feature H was constructed and appended to each atom node feature as $\langle V,H\rangle =\left\{\text{Concat}\left(v_{i},H\right)|v_{i}\in V\right\}$ using SMILES encoding and molecule properties (Table S7, Supporting Information). This hierarchical representation allowed to capture both local atomic information and global molecular characteristics. The PROTAC SMILES were first mapped into frequency encoding S, with a maximum allowed string length of |S| using the lead‐like molecules encoding table which captures the statistical chemical composition and structural motifs present in the molecule. This table collected by Li et al^[^ ^18^ ^]^ encoded the 39 most frequent characters from 1 to 39, based on counts from the ZINC database^[^ ^55^ ^]^. Nine molecule properties were directly obtained from the PROTAC table in PROTAC‐DB^[^ ^32^ ^]^, denoted as a real‐valued vector R. To ensure consistent scaling across diverse properties, these values were standardized using mean and standard deviation normalization. Thus, the global feature $H=\text{Concat}\left(S,R\right)$ was constructed, where $H\in R^{|S|+9}$, and $\langle V,H\rangle \in R^{\left|V\right|\times D_{t}}$, where $D_{t}=9+\left|S\right|+9$, leading to the input PROTAC feature denoted as $X_{t}^{0}=G\left(\left\langle V,H\right\rangle ,E\right)$. The hierarchical encoding enabled the model to learn both detailed atomic interactions and the broader influence of molecular properties on PROTAC degradation activity.

Finally, the hierarchical graph representation of PROTAC molecules was learned through a two‐layer GCN module. GCNs extend convolutional operations to non‐Euclidean domains, enabling the processing of irregular graph structures. In this process, atom feature matrices were updated by aggregating information from adjacent atoms and their associated chemical bonds. This propagation mechanism inherently captured the substructure information of PROTAC molecules, preserving essential features for subsequent explicit learning of substructure interactions with POI and E3 ligase. The hierarchical PROTAC encoder can be formally expressed as:

(1) $$X_{t}^{i+1}=\sigma \left({\text{GCN}}^{*}\left(\tilde{A},W_{g}^{i},b_{g}^{i},X_{t}^{i}\right)\right)$$

where GCN*(·) is this modified GCN to adopt edge attributes, $W_{g}^{i}$ and $b_{g}^{i}$ are the GCN's layer‐specific learnable weight matrix and bias vector; $\tilde{A}$ is the adjacency matrix with added self‐loops in molecular graph G; $X_{t}^{i}$ is the *i*‐th hidden representation; and σ(·) denotes a non‐linear activation function.

*Structural Encoders for POIs and E3 Ligases*

Due to the scarcity of crystal structures for PROTAC‐mediated ternary complexes, it was challenging to identify the accurate binding pocket of POI and E3 ligase. Moreover, the sequence data for POIs and E3 ligases was readily available. Therefore, the abundance of protein sequence data was leveraged and the entire protein structure was considered. As illustrated in Figure 8b, the structural protein encoder consists of a protein language model (PLM) embedder and a linear adapter, which introduced crucial structural information into protein embedding for protein sequence data without requiring explicit protein structures as input. This approach, inspired by ESM‐S^[^ ^30^, ^31^ ^]^, allowed to represent proteins structurally without relying on explicit 3D structures. The structural encoder offered a powerful alternative when direct structural data was limited, enabling the model to learn and utilize the inherent structural information encoded within protein sequences.

Specifically, the original data for POI and E3 ligase derived from PROTAC‐DB were their UniProt IDs^[^ ^56^ ^]^. The protein amino acid sequence $P_{p}=\left[r_{p1},r_{p2},\text{…}\right]$ for POI and $P_{e}=\left[r_{e1},r_{e2},\text{…}\right]$ for E3 ligase was first retrieved, where each *r*_*i*_ corresponds to the type of the *i*‐th residue and Figure S2 (Supporting Information) provided the length distribution of all proteins. For each protein of POIs and E3 ligases, a residue protein view and then truncate the protein was constructed to a maximum allowed length of 1022^[^ ^30^ ^]^ using the TorchDrug package. The PLM embedder **ϕ** was built upon the ESM‐S^[^ ^31^ ^]^ model, which used remote homology detection to distill structural information into ESM^[^ ^30^ ^]^, with **ϕ**_*p*_ for POI and **ϕ**_*e*_ for E3 ligase. In the PLM embedder module, the protein sequence data was processed into the token representation via several self‐attention and feed‐forward networks, and then the sequence representation of a fixed length **θ_p_** = 1280 was generated in an averaging way. Finally, The input POI and E3 ligase data are denoted as $X_{p}\in R^{\theta_{p}}$, $X_{e}\in R^{\theta_{p}}$, respectively. The structural POI and E3 ligase encoders are described as follows:

(2) $$X_{p}=\sigma \left(\phi_{p}\left(W_{p},b_{p},P_{p}\right)\right)$$

(3) $$X_{e}=\sigma \left(\phi_{e}\left(W_{e},b_{e},P_{e}\right)\right)$$

where **W**_*p*_, **W**_*e*_ and **b**_*p*_, **b**_*e*_ are the learnable weight matrices (adopters) and bias vectors after the PLM embedder module. **X**_*p*_ is the structural embedding of the POI and **X**_*e*_ is the structural embedding of the E3 ligase.

*Ternary Attention Network for Interaction Learning*

To capture the complex interplay among POIs, PROTAC molecules, and E3 ligases, a novel ternary attention network (TAN) tailored was introduced for the PROTAC system. Traditional attention^[^ ^57^, ^58^, ^59^ ^]^ mechanisms often focus on pairwise interactions, but PROTACs inherently involve three distinct entities. The TAN, illustrated in Figure 8c, extends this concept to model the intricate relationships among all three simultaneously through a ternary attention map that quantified interaction intensities and a fusion layer that integrates these interactions into a unified representation. Previous Bilinear Attention Networks (BAN)^[^ ^58^ ^]^ which are designed for Visual Question Answering (VQA) were generalized to a unified network. Interestingly, BAN could be viewed as a subset of the TAN method, as discussed in Supporting Information (Experimental Section Generality of TAN), validating the integrity and enhancing the generality of this approach.

TAN comprises two essential components: i) a ternary attention map to capture interaction intensity, and ii) a ternary fusion layer over the attention map to extract joint POI‐PROTAC‐E3 representation. TAN enabled simultaneous modeling of interactions among all three entities, yielding comprehensive interaction data. This network was particularly well‐suited for PROTAC system modeling, as it facilitated concurrent learning of substructure interactions, a crucial aspect in understanding PROTAC‐mediated protein degradation. This simultaneous modeling of multi‐entity interactions allowed the model to learn intricate substructure relationships, leading to improved prediction accuracy and greater interpretability of PROTAC‐mediated protein degradation.

Given the learned hidden representation of POI, PROTAC molecule and E3 ligase $X_{p}\in R^{D_{p}\times \alpha }$, $X_{t}\in R^{D_{t}\times \beta }$, and $X_{e}\in R^{D_{e}\times \gamma }$ after separate hierarchical molecular and structural protein encoders, where $\alpha =|\{x_{p}^{i}\}|$, $\beta =|\{x_{t}^{i}\}|$, and $\gamma =|\{x_{e}^{i}\}|$ denote the number of encoded substructures in POI, PROTAC molecule, and E3 ligase, respectively. Their ternary interaction can obtain a three‐dimension ternary attention map $A\in R^{\alpha \times \beta \times \gamma }$:

(4) $$A=\left[\left(1\cdot q^{⊤}\right)⊙\sigma \left(X_{p}^{⊤}\cdot U\right)\right]★\sigma \left(X_{t}^{⊤}\cdot W\right)\cdot \sigma \left(V^{⊤}\cdot X_{e}\right)$$

where $U\in R^{D_{p}\times \kappa }$, $W\in R^{D_{t}\times \kappa }$, $V\in R^{D_{e}\times \kappa }$ represent learnable weight matrices for POI, PROTAC, and E3 ligase representations, respectively. $q\in R^{\kappa }$ denotes a learnable weight vector, $1\in R^{\alpha }$ is a constant vector of ones. ⊙ represents the Hadamard (element‐wise) product, ⋆ signifies the Einstein summation convention (einsum)^[^ ^60^ ^]^, an efficient computational method that simplifies tensor operations, specially *ik*, *jk* → *ijk*, and · denotes standard matrix multiplication. The elements of A quantify the interaction intensity among (POI, PROTAC, E3) substructural triplets, correlating with potential binding sites and molecular substructures. For a more intuitive understanding of the ternary interaction, an individual element $A_{i,j,k}$ from Equation (4) can be expressed as:

(5) $$A_{i,j,k}=q^{⊤}\left[\sigma \left(U^{⊤}x_{p}^{i}\right)⊙\sigma \left(W^{⊤}x_{t}^{j}\right)⊙\sigma \left(V^{⊤}x_{e}^{k}\right)\right]$$

where $x_{p}^{i}$, $x_{t}^{j}$, and $x_{e}^{k}$ represent the *i*‐th, *j*‐th, and *k*‐th columns of **X**_*p*_, **X**_*t*_, and **X**_*e*_, respectively. These columns correspond to the substructural representations of POI, PROTAC molecule, and E3 ligase. The ternary interaction could be conceptualized as first projecting representations $x_{p}^{i}$, $x_{t}^{j}$, and $x_{e}^{k}$ to a feature space with weight matrices **U**, **W**, and **V**, then learning the ternary attention map based on operation product of these projections and the weight vector **q**. This formulation of ternary interactions enabled the interpretation of how substructural triplets contribute to the prediction outcomes, thereby enhancing the model's explainability.

For the subsequent classification step, a ternary fusion layer over the attention map A was introduced to derive the joint representation 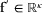. The *k*‐th element of 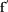 is computed as follows:

(6)

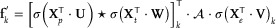

where ${\left[\sigma \left(X_{p}^{⊤}\cdot U\right)★\sigma \left(X_{t}^{⊤}\cdot W\right)\right]}_{k}\in R^{\alpha \times \beta }$, $\sigma {\left(X_{e}^{⊤}\cdot V\right)}_{k}\in R^{\gamma }$, reminded that ⋆ denotes the einsum^[^ ^60^ ^]^ *ik*, *jk* → *ijk*. Notably, no new learnable parameters are introduced at this layer, weight matrices **U**, **W**, and **V** are shared with the preceding ternary interaction layer, reducing parameter count and mitigating overfitting risk. Furthermore, the single ternary interaction was extended to a multi‐head formulation by computing multiple ternary interaction maps. The final joint representation vector was obtained by summing individual heads. As the weight matrices **U**, **W**, and **V** were shared, each additional head introduces only one new weight **q**, ensuring parameter efficiency. In this experiments, the multi‐head interaction performs better than a single one. Lastly, a pooling operation was applied on the joint representation vector to obtain a compact feature 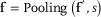, where Pooling(·) represents a 1D average pooling operation with stride *s*, reducing the dimensionality from 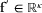 to $f\in R^{\kappa /s}$.

In the final stage of the model, the joint representation **f** is processed through a classifier. This classifier consisted of a multi‐layer perceptron (MLP) classification layer, denoted as ψ_*MLP*_, followed by a LogSoftmax activation function. The degradation prediction result **p** is computed as follows:

(7) $$p=\text{LogSoftmax}\left(\psi_{MLP}\left(f\right)\right)$$

where ψ_*MLP*_ is composed of two fully connected layers interspersed with a nonlinear activation function.

The novel ternary attention mechanism enabled the model to explicitly capture and learn the intricate substructure interactions among POI, PROTAC molecule, and E3 ligase. By leveraging the learned ternary attention map, the contribution of individual substructures to the final outcome can be visualized and quantified. This approach not only aids in elucidating the underlying binding mechanisms but also significantly enhances the model's interpretability, providing valuable insights into the complex interplay of PROTAC‐mediated protein degradation.

### MD Simulation

The crystal structures of CDK4 (PDB ID: 7SJ3) and VHL (PDB ID: 5T35) were preprocessed using the Protein Preparation Wizard in Maestro (Schrödinger 2019‐2). Ligands bound to CDK4 (palbociclib) and VHL (PubChem Compound CID: 129900323) were optimized through the LigPrep module. Molecular docking of ligands to their respective targets was performed using Glide XP mode to generate protein‐ligand complexes. PROTAC structures were designed in ChemDraw and exported as SMILES files. Ternary complexes (CDK4‐PROTAC‐VHL) were constructed in MOE 2019.01 following the 4B method proposed by Drummond et al.^[^ ^61^ ^]^. The ternary complexes served as initial conformations for molecular dynamics simulations performed in AMBER24. Ligand charges were assigned using the RESP methodology with the GAFF2 force field for ligands and AMBER19SB for proteins. Systems were solvated in a TIP3P water box with a 10 Åbuffer and subjected to sequential equilibration: 70,000 steps of energy minimization (steepest descent followed by conjugate gradient), 200 ps heating to 310 K under NVT ensemble, and 1 ns restrained equilibration under NPT ensemble. Production simulations consisted of 500 ‐ns unrestrained MD under NPT conditions (310 K, 1 atm). After completing the simulation, the resulting trajectory was analyzed, which included the following content:

1. Calculating binding free energy using the MM‐GBSA method, processing the last 50 ns of the equilibrated trajectory with MMPBSA.py.MPI.
2. Characterizing protein‐protein interactions by measuring the distance between CDK4 (residue Arg101) and VHL (residue Tyr98);
3. Evaluating the binding stability of the ligand relative to the protein binding site through Root Mean Square Deviation (RMSD);
4. Assessing the likelihood of PROTAC‐induced complex degradation using lysine solvent‐accessible surface areas (SASA)^[^ ^62^ ^]^.

All analysis results were statistically computed using the cpptraj module in AmberTools24.

### Experiment Settings

*Metrics*

To evaluate the performance of the model and compare it with existing methods, several standard metrics were employed:

1. Accuracy: This metric provided the overall correct prediction rate, offering a general measure of the model's performance.
(8) $$\text{Accuracy}=\frac{\text{TP}+\text{TN}}{\text{TP}+\text{TN}+\text{FP}+\text{FN}}$$
  where TP, TN, FP, and FN represent True Positives, True Negatives, False Positives, and False Negatives, respectively.
2. Area Under the Receiver Operating Characteristic curve (AUROC): This metric assesses the model's ability to distinguish between classes across various threshold settings, providing a comprehensive view of classification performance.
(9) $$\text{AUROC}=\int_{0}^{1}\text{TPR}\left({\text{FPR}}^{-1}\left(t\right)\right)dt$$
  where TPR = TP / (TP + FN) is the True Positive Rate and FPR = FP / (FP + TN) is the False Positive Rate.
3. F1 score: The F1 score was the harmonic mean of precision and recall, offering a balanced measure of the model's performance, particularly useful in scenarios where class imbalance may be present.
(10) $$\text{F1}=2\cdot \frac{\text{Precision}\cdot \text{Recall}}{\text{Precision}+\text{Recall}}$$
  where Precision = TP / (TP + FP) and Recall = TP / (TP + FN).

These metrics collectively provided a robust evaluation for assessing the efficacy of our model in predicting degradation outcomes.

*Baselines*

PROTAC‐STAN was compared with the following three models for PROTAC degradation prediction: 1) Two classic machine learning methods, support vector matching (SVM)^[^ ^33^ ^]^ and random forest (RF)^[^ ^36^ ^]^ by employing the auto cross‐covariance (ACC) features for the POI and E3 ligase, and the molecular access system (MACCS) keys^[^ ^34^ ^]^ or Morgan fingerprints^[^ ^35^ ^]^ for the PROTAC molecule following the approach described by Li et al.^[^ ^18^ ^]^ 2) DeepPROTACs^[^ ^18^ ^]^ that predict PROTAC degradation using GNN to encode molecular graph of ligands and generated pockets of POI and E3 ligase, along with RNN to encode linker SMILES. The learned three features were combined with a simple concatenation to predict final degradation. For the above degradation prediction models, the SVM and RF models follow the recommended parameter settings as described in the reference paper^[^ ^18^ ^]^, and the results reported in the original DeepPROTACs paper were used since their dataset was not publicly available.

*Implementation*

PROTAC‐STAN was implemented in python 3.11 and pytorch 2.1.0, along with functions from pytorch geometric 2.5.1, rdkit 2023.9.2, scikit‐learn 1.2.2, pandas 2.1.1, numpy 1.26.4, and biopython 1.83. The training was finished under an Nvidia GeForce RTX 3090 GPU. The batch size was set to be four and the Adam optimizer was used with a learning rate of 0.0005. The model was allowed to run for most 100 epochs with an early stop patience set to 30. The best‐performing model was selected at the epoch giving the best AUROC score. In the ablation experiments, the same hyperparameter configuration was used for all experiments to ensure comparability. Each experiment was repeated ten times, and the results reported in the table were those from the epoch that achieved the best AUROC score. For the Raw approach, the SMILESNet encoder^[^ ^18^ ^]^ and the Ngrams encoder^[^ ^14^ ^]^ were implemented using their original configurations. The PROTAC feature encoder consisted of one linear layer with hidden dimensions [146, 64], two GCN layers with hidden dimensions [128, 128], and an edge dimension of three. The POI and E3 ligase feature encoders consist of a PLM embedder with an embedding dimension of 1280 and two linear layers with hidden dimensions [128, 64]. The maximum allowed number of SMILES characters for PROTAC was set to 128, and the maximum permitted protein sequence length for POIs and E3 ligases is 1022. In the ternary attention module, two attention heads were employed to provide better interpretability. The number of hidden neurons in the MLP classifier is set to 64. Part of the illustrations in this article were created using ML Visuals material.

## Conflict of Interest

The authors declare no conflict of interest.

## Author Contributions

Z.‐C., and C.‐G., contributed equally to this work. Z.C. and C.G. conceived the idea, prepared the data, developed the theoretical formalism, constructed the model and conducted experiments, and wrote the manuscript; Z.C., S.T., and X.W. investigated the PROTAC degradation developments, verified the analytical methods; Y.L. helped write and revise the manuscript; Z.C. and M.H. analyzed the visualizations; R.L. and S.S. contributed to the interpretability experiments. C.G, C.H., X.Y., and H.L. supervised the project, and revised the manuscript together with P.H. All authors discussed the results and contributed to the final manuscript.

## Supporting information

Supporting Information

## Data Availability

All data are publicly available and can be accessed at http://cadd.zju.edu.cn/protacdb/ for PROTAC‐DB, https://github.com/PROTACs/PROTAC‐STAN for PROTAC‐fine dataset. All code of PROTAC‐STAN is available at https://github.com/PROTACs/PROTAC‐STAN.
