# Supplementary material for: Interpretable PROTAC Degradation Prediction With Structure‐Informed Deep Ternary Attention Framework
Source: Adv Sci (Weinh). 2025 Sep 30;12(47):e08138. doi: 10.1002/advs.202508138 (PMC12713099; doi:10.1002/advs.202508138)
Supplement: Supplementary file 1 — Supporting Information [file ADVS-12-e08138-s001.pdf]

Supporting Information for Interpretable PROTAC degradation prediction with structure-informed deep ternary attention framework

Zhenglu Chen[†] *Chunbin Gu**[†] *Shuoyan Tan* *Xiaorui Wang* *Yuquan Li* *Mutian He* *Ruiqiang Lu* *Shijia Sun* *Chang-Yu Hsieh** *Xiaojun Yao** *Huanxiang Liu** *Pheng-Ann Heng*

[†] *These authors contributed equally to this work.*

This PDF file includes:

Materials and Methods
Tables S1 to S11
Figures S1 to S8
Caption for Data S1

Other Supporting infromation for this manuscript:

Data S1

Contents

S1 Materials and Methods **3**
S1.1 Model and Methodology . . . 3
S1.1.1 Notations and Definitions . . . 3
S1.1.2 Generality of TAN . . . 3
S1.1.3 PROTAC Design and MD Validation . . . 4
S1.1.4 Correlation Analysis in Case Study . . . 7
S1.2 Implementation and Experimental Setup . . . 7
S1.2.1 Hyperparemeters and Configurations . . . 7
S1.2.2 Computational Efficiency Comparison . . . 7
S1.2.3 Residue Selection and Position Assessment . . . 9
S1.2.4 Top Residue Statistics . . . 11
S1.2.5 Sequence Length Statistics of POI/E3 ligase . . . 11
S1.2.6 Molecular Properties in Global Feature . . . 12
S1.2.7 Implicit DC_{50} and D_{max} from Experimental Descriptions . . . 12
S1.3 Ablation and Performance Analysis . . . 14
S1.3.1 Component-wise Ablation on PROTAC-STAN . . . 14
S1.3.2 SMILES Length and ESM-2 Ablation . . . 14
S1.3.3 Prospective Integration of ESM-S with AlphaFold3 . . . 15
S1.3.4 TAN Module Ablation Results . . . 15
S1.3.5 Degradation Performance: PROTAC-fine vs. PROTAC-DB 3.0 . . . 15
S1.3.6 Degradation Performance with Rigid Linkers . . . 17
S1.4 Supplementary Visualizations . . . 17
S1.4.1 3D Attention Map . . . 17
S1.4.2 2D Attention Map . . . 17
S1.4.3 2D PROTAC Molecule . . . 18
S1.4.4 2D Interaction and 3D Complex . . . 18

S2 Caption for Data S1 **22**

S1 Materials and Methods

S1.1 Model and Methodology

S1.1.1 Notations and Definitions

The major notations including the professional abbreviations and mathematical symbols used in this paper are provided in Table S1.

Table S1: **Major notations.**

Notation	Definition
PROTAC	PROteolysis TArgeting Chimeras
POI	Protein Of Interest
TPD	Targeted Protein Degradation
UPS	Ubiquitin-Proteasome System
DC_{50}	Half-maximal degradation concentration
D_{max}	Maximum level of protein degradation
$\mathcal{P}_p$	A POI
$\mathcal{G}$	A PORTAC molecule
l	Binary degradation label
$\mathcal{P}_e$	An E3 ligase
$\mathcal{V}$	Node information of PROTAC molecule
$\mathcal{E}$	Edge information of PROTAC molecule
$\mathbf{X}_t \in \mathbb{R}^{D_t \times \beta}$	Encoded PROTAC feature representation
$\sigma(\cdot)$	Non-linear activation function
$\mathbf{W}^i$	Learnable weight matrices
$\mathbf{b}^i$	Bias vectors
$\mathbf{X}_p \in \mathbb{R}^{D_p \times \alpha}$	Encoded POI feature representation
$\mathbf{X}_e \in \mathbb{R}^{D_e \times \gamma}$	Encoded E3 ligase feature representation
$\mathbf{U} \in \mathbb{R}^{D_p \times \kappa}$	Learnable weight matrix for POI
$\mathbf{W} \in \mathbb{R}^{D_t \times \kappa}$	Learnable weight matrix for PROTAC
$\mathbf{V} \in \mathbb{R}^{D_e \times \kappa}$	Learnable weight matrix for E3 ligase
$\mathbf{q} \in \mathbb{R}^{\kappa}$	Learnable vector
$\odot$	Hadamard (element-wise) product
$\star$	Einstein summation convention, $ik, jk \rightarrow ijk$
$\cdot$	Matrix multiplication
$\mathbf{f}' \in \mathbb{R}^{\kappa}$	Joint POI-PROTAC-E3 representation
p	Degradation prediction

S1.1.2 Generality of TAN

Review of TAN: Given the representation of three entities inputs $\mathbf{X}_a \in \mathbb{R}^{D_a \times \alpha}$, $\mathbf{X}_b \in \mathbb{R}^{D_b \times \beta}$, and $\mathbf{X}_c \in \mathbb{R}^{D_c \times \gamma}$, where $\alpha = |\{x_a^i\}|$, $\beta = |\{x_b^i\}|$, and $\gamma = |\{x_c^i\}|$ denote the number of substructures in three entities. We define TAN as a function of the inputs from three entities, parameterized by a ternary attention map, as follows:

$$\mathbf{f} = \mathbf{TAN}(\mathbf{X_a}, \mathbf{X_b}, \mathbf{X_c}; \mathcal{A}) \tag{S1}$$

Ternary attention map. The ternary interaction can obtain a three-dimension ternary attention map $\mathcal{A} \in \mathbb{R}^{\alpha \times \beta \times \gamma}$:

$$\mathcal{A} = \left[\left(\mathbf{1} \cdot \mathbf{q}^{\top}\right) \odot \sigma\left(\mathbf{X}_a^{\top} \cdot \mathbf{U}\right)\right] \star \sigma\left(\mathbf{X}_b^{\top} \cdot \mathbf{W}\right) \cdot \sigma\left(\mathbf{V}^{\top} \cdot \mathbf{X}_c\right), \tag{S2}$$

where $\mathbf{U} \in \mathbb{R}^{D_a \times \kappa}$, $\mathbf{W} \in \mathbb{R}^{D_b \times \kappa}$, $\mathbf{V} \in \mathbb{R}^{D_c \times \kappa}$ represent learnable weight matrices for three representations, respectively. $\mathbf{q} \in \mathbb{R}^{\kappa}$ denotes a learnable weight vector, $\mathbf{1} \in \mathbb{R}^{\alpha}$ is a constant vector of ones. $\odot$ represents the Hadamard (element-wise) product, $\star$ signifies the Einstein summation convention (einsum)[1], specially $ik, jk \rightarrow ijk$, and $\cdot$ denotes standard matrix multiplication. The elements of $\mathcal{A}$ quantify the

interaction intensity among substructural triplets. An individual element $\mathcal{A}_{i,j,k}$ from Equation S2 can be expressed as:

$$\mathcal{A}_{i,j,k} = \mathbf{q}^\top \left[\sigma(\mathbf{U}^\top \mathbf{x}_a^i) \odot \sigma(\mathbf{W}^\top \mathbf{x}_b^j) \odot \sigma(\mathbf{V}^\top \mathbf{x}_c^k)\right], \tag{S3}$$

where $\mathbf{x}_a^i$, $\mathbf{x}_b^j$, and $\mathbf{x}_c^k$ represent the i-th, j-th, and k-th columns of $\mathbf{X}_a$, $\mathbf{X}_b$, and $\mathbf{X}_c$, respectively.

Ternary fusion. Next, we introduce a ternary fusion layer over the attention map $\mathcal{A}$ to derive the joint representation $\mathbf{f} \in \mathbb{R}^\kappa$. The k-th element of $\mathbf{f}$ is computed as follows:

$$\mathbf{f}_k = \left[\sigma\left(\mathbf{X}_a^\top \cdot \mathbf{U}\right) \star \sigma\left(\mathbf{X}_b^\top \cdot \mathbf{W}\right)\right]_k^\top \cdot \mathcal{A} \cdot \sigma\left(\mathbf{X}_c^\top \cdot \mathbf{V}\right)_k, \tag{S4}$$

where $\left[\sigma\left(\mathbf{X}_a^\top \cdot \mathbf{U}\right) \star \sigma\left(\mathbf{X}_b^\top \cdot \mathbf{W}\right)\right]_k \in \mathbb{R}^{\alpha\times\beta}$, $\sigma\left(\mathbf{X}_c^\top \cdot \mathbf{V}\right)_k \in \mathbb{R}^\gamma$. Furthermore, we extend the single ternary interaction to a multi-head formulation by computing multiple ternary interaction maps. The final joint representation vector is obtained by summing individual heads.

Simplified to BAN: Bilinear Attention Network (BAN)[2] is designed for Visual Question Answering, handling two multi-channel inputs: image features $\mathbf{X} \in \mathbb{R}^{N\times\rho}$ and text features $\mathbf{Y} \in \mathbb{R}^{M\times\phi}$. Similar to TAN, BAN can be defined as follows:

$$\mathbf{f} = \mathbf{BAN}(\mathbf{X}, \mathbf{Y}; \mathcal{A}) \tag{S5}$$

From TAN, let $\mathbf{X}_a \Leftarrow \mathbf{X}$, $\mathbf{X}_b \Leftarrow \mathbb{I}$, and $\mathbf{X}_c \Leftarrow \mathbf{Y}$, where $\mathbb{I} \in \mathbb{R}^{D\times 1}$ is a vector of ones, we then obtain a two-dimension attention map $\mathcal{A} \in \mathbb{R}^{\rho\times\phi}$ as follows:

$$\begin{aligned}\mathcal{A} &= \left[\left(\mathbf{1}\cdot\mathbf{q}^\top\right) \odot \sigma\left(\mathbf{X}_a^\top \cdot \mathbf{U}\right)\right] \star \sigma\left(\mathbf{X}_b^\top \cdot \mathbf{W}\right) \cdot \sigma\left(\mathbf{V}^\top \cdot \mathbf{X}_c\right) \\ &= \left[\left(\mathbf{1}\cdot\mathbf{q}^\top\right) \odot \sigma\left(\mathbf{X}^\top \cdot \mathbf{U}\right)\right] \star \sigma\left(\mathbb{I}^\top \cdot \mathbf{W}\right) \cdot \sigma\left(\mathbf{V}^\top \cdot \mathbf{Y}\right) \\ &= \left[\left(\mathbf{1}\cdot\mathbf{q}^\top\right) \odot \sigma\left(\mathbf{X}^\top \cdot \mathbf{U}\right)\right] \cdot \sigma\left(\mathbf{V}^\top \cdot \mathbf{Y}\right)\end{aligned} \tag{S6}$$

where $\mathbf{U} \in \mathbb{R}^{M\times\kappa}$, $\mathbf{W} \in \mathbb{R}^{D\times\kappa}$, $\mathbf{V} \in \mathbb{R}^{N\times\kappa}$ represent learnable weight matrices for three representations, respectively. $\mathbf{q} \in \mathbb{R}^\kappa$ denotes a learnable weight vector, $\mathbf{1} \in \mathbb{R}^\rho$ is a constant vector of ones. An individual element $\mathcal{A}_{i,j}$ from Equation S6 can be expressed as:

$$\mathcal{A}_{i,j} = \mathbf{q}^\top \left[\sigma(\mathbf{U}^\top \mathbf{x}^i) \odot \sigma(\mathbf{V}^\top \mathbf{y}^j)\right], \tag{S7}$$

where $\mathbf{x}^i$, $\mathbf{y}^j$ represent the i-th, j-th columns of $\mathbf{X}$, $\mathbf{Y}$.

Similar to the ternary fusion, we can derive the joint representation of BAN, denoted as $\mathbf{f} \in \mathbb{R}^\kappa$, using the attention map obtained in Equation S6. The k-th element of $\mathbf{f}$ is computed as follows:

$$\begin{aligned}\mathbf{f}_k &= \left[\sigma\left(\mathbf{X}_a^\top \cdot \mathbf{U}\right) \star \sigma\left(\mathbf{X}_b^\top \cdot \mathbf{W}\right)\right]_k^\top \cdot \mathcal{A} \cdot \sigma\left(\mathbf{X}_c^\top \cdot \mathbf{V}\right)_k \\ &= \left[\sigma\left(\mathbf{X}^\top \cdot \mathbf{U}\right) \star \sigma\left(\mathbb{I}^\top \cdot \mathbf{W}\right)\right]_k^\top \cdot \mathcal{A} \cdot \sigma\left(\mathbf{Y}^\top \cdot \mathbf{V}\right)_k \\ &= \left[\sigma\left(\mathbf{X}^\top \cdot \mathbf{U}\right)\right]_k^\top \cdot \mathcal{A} \cdot \sigma\left(\mathbf{Y}^\top \cdot \mathbf{V}\right)_k\end{aligned} \tag{S8}$$

where $\left[\sigma\left(\mathbf{X}^\top \cdot \mathbf{U}\right)\right]_k \in \mathbb{R}^\rho$, $\sigma\left(\mathbf{Y}^\top \cdot \mathbf{V}\right)_k \in \mathbb{R}^\phi$.

Generally, TAN can be applied to any scenario involving the interplay of three entities, providing nuanced insights into multi-entity interactions. This leads to more accurate predictions and enhanced interpretability of results. In the case of TAN and BAN, our proposed TAN offers a unified framework for attention networks with multiple inputs. Looking ahead, we may extend the Ternary Attention Network to an N-ary Attention Network, such as a quaternary attention network, in specific scenarios.

S1.1.3 PROTAC Design and MD Validation

We selected Cyclin-dependent kinase 4 (CDK4) as the target for our case study. Specifically, we chose the known CDK4 inhibitor, Palbociclib, as the warhead component of the PROTAC, and utilized a von

Hippel-Lindau (VHL)-binder as the E3 ligase ligand. From the PROTAC-fine dataset, we identified nine entries where the POI is 'CDK4' and the E3 ligase is 'VHL'. Among these, we focused on three entries with 'True' labels, which featured three distinct PROTACs (with one unique warhead and two different E3 ligands) and seven different linkers. These were used as references for the construction of our PROTACs.
Furthermore, we identified a total of 128 CDK-targeted entries within the dataset, which included 51 different linkers. After excluding the seven linkers selected under the CDK4 and VHL conditions, we were left with 44 linker candidates. Using RDKit, we filtered out erroneous linkers using RDKit, resulting in 40 valid candidates. Based on this pool, we employed Chemdraw software to construct 13 distinct PROTACs, considering the linker length, type, and flexibility. The final list of PROTAC candidates is detailed in Table S2. **Prediction** are predicted results from our PROTAC-STAN model: 0 indicates low degradation capability, while 1 indicates high degradation capability. **MM-GBSA**, the Molecular Mechanics-Generalized Born Surface Area method extracted the final 50 ns equilibrium segment from the overall 500 ns full-length trajectory to calculate the binding free energy between the CDK4 and VHL proteins (unit: kcal/mol). More negative values indicate lower binding free energy and stronger binding affinity. NA indicates that the system collapsed during the simulation, and the protein-protein binding affinity was not computed.

Table S2: **Built PROTACs and validation results.**

ID	PROTAC	Flexibility	Prediction (Score)	MM-GBSA
1	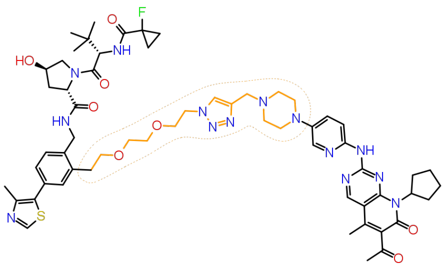	0.53	1 (0.8942)	-39.25
2	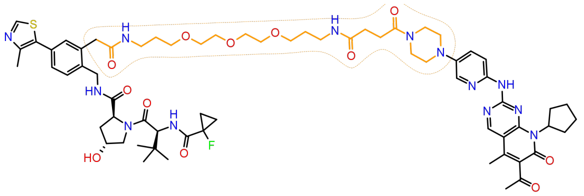	0.75	1 (0.9985)	-24.31
3	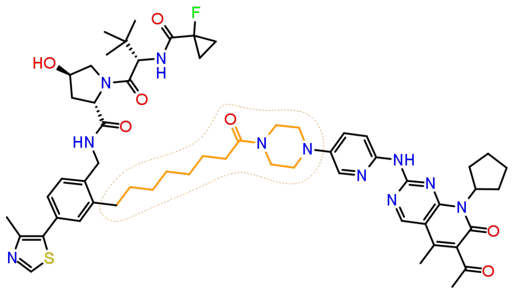	0.75	1 (0.5882)	-22.86
4	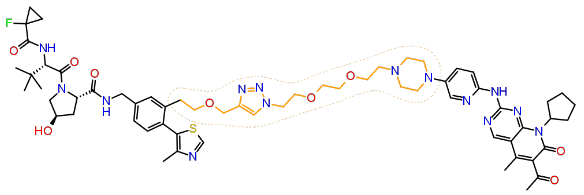	0.61	1 (0.8865)	-22.03

(Continued on next page)

(Continued from previous page)

ID	PROTAC	Flexibility	Prediction (Score)	MM-GBSA
5	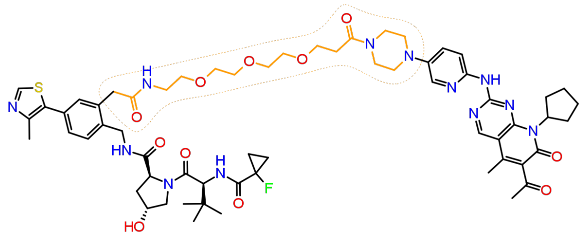	0.75	1 (0.9954)	-20.00
6	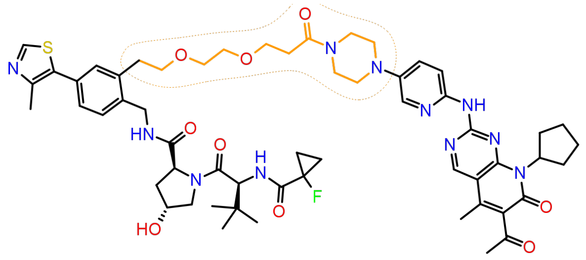	0.78	1 (0.9863)	-19.12
7	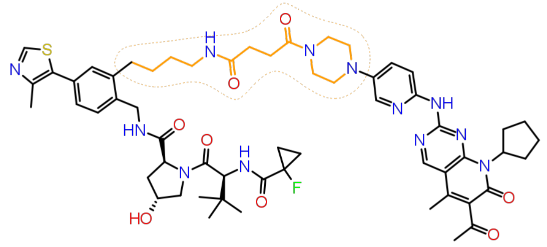	0.64	1 (0.7210)	-17.96
8	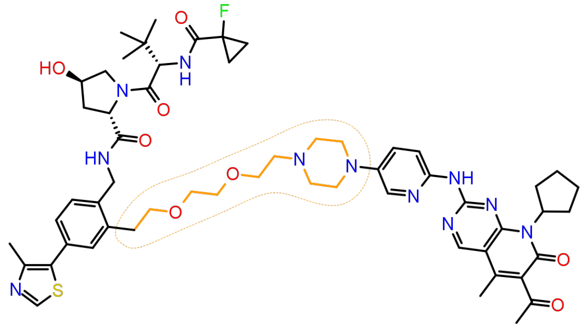	0.75	0 (0.4928)	-13.47
9	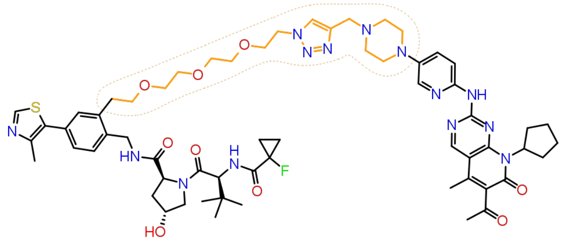	0.61	1 (0.9882)	-11.23
10	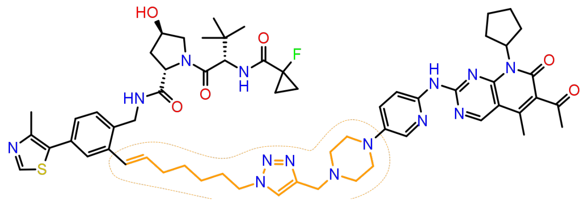	0.38	1 (0.9978)	-9.76

(Continued on next page)

(Continued from previous page)

ID	PROTAC	Flexibility	Prediction (Score)	MM-GBSA
11	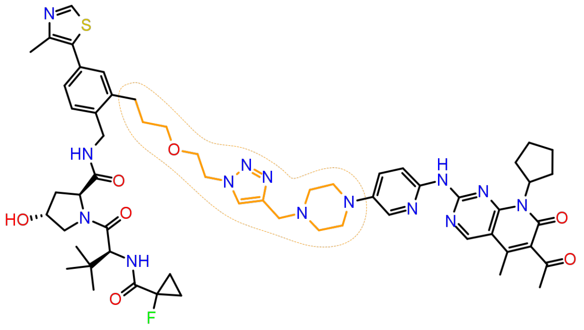	0.42	0 (0.2172)	-2.34
12	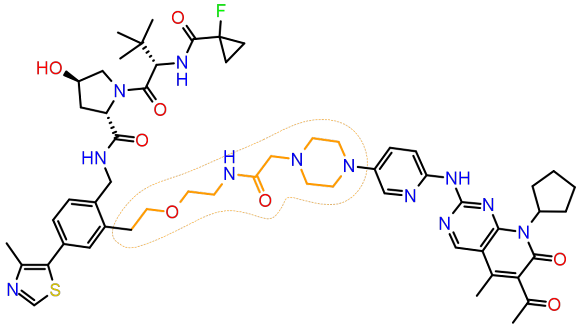	0.56	0 (0.2517)	NA
13	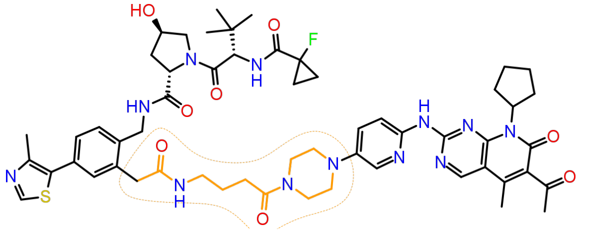	0.56	0 (0.2500)	NA

S1.1.4 Correlation Analysis in Case Study

We conducted a correlation analysis between PROTAC-STAN's prediction score and the corresponding MM-GBSA binding free energy. As shown in Figure S1, a negative correlation trend is observed, suggesting an inverse relationship between model predictions and physical stability. Furthermore, we also calculated:

- Pearson correlation coefficient: r = –0.660 (p = 0.0141), indicating a statistically significant linear correlation.
- Spearman rank correlation: ρ = –0.514 (p = 0.0721), reflecting a moderately strong monotonic association.

These results indicate that the predictions made by PROTAC-STAN are generally aligned with the physical stability profiles inferred from MD simulations, thus supporting the model's practical reliability.

S1.2 Implementation and Experimental Setup

S1.2.1 Hyperparemeters and Configurations

Here, we demonstrate the hyperparameters and training configuration used in our methods as in Table S3.

S1.2.2 Computational Efficiency Comparison

Table S4 added SVM and Random Forest (RF) to the analysis to provide a more comprehensive assessment of real-world deployment costs. As expected, due to their relatively simple and linear computational nature, both SVM and RF exhibit extremely fast training and inference times. These models can

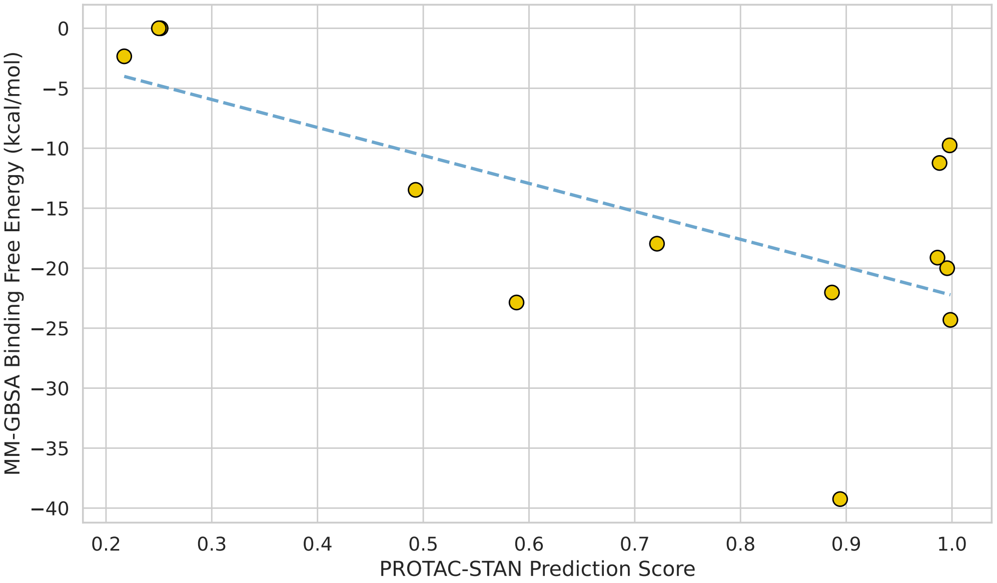

Figure S1: **Correlation between PROTAC-STAN Prediction Scores and MM-GBSA Binding Free Energy.** Pearson correlation coefficient: r = –0.660 (p = 0.0141), Spearman rank correlation: ρ = –0.514 (p = 0.0721).

Table S3: **Summary of hyperparameters and configurations.**

Parameter	Value	Description
Model		
Max SMILES length	128	Maximum number of PROTAC SMILES characters
PROTAC feature	146	Dimension of input PROTAC features
PROTAC hidden state	128	Dimension of PROTAC hidden states
PROTAC embedding	128	Dimension of PROTAC embeddings
PROTAC edge	3	Dimension of PROTAC graph edges
Max protein length	1022	Maximum number of amino acids of input POIs/E3 ligases
Sequence embedding	1280	Dimension of POI/E3 Sequence embedding
POI/E3 hidden state	128	Dimension of POI/E3 hidden states
POI/E3 output	64	Dimension of POI/E3 outputs
Fusion feature	192	Dimension of fusion features
Multiheads	2	Number of attention heads
Dropout	[0.2, 0.2, 0.2]	Dropout ratio of each input features
Joint feature embedding	192	Dimension of joint POI-PROTAC-E3 feature embedding
Hidden state	64	Dimension of joint feature hidden states
Class	2	Number of classification classes
Training		
Learning rate	0.0005	Learning rate for the optimizer
Batch size	4	Number of samples per step
Epochs	100	Number of times to iterate over the entire dataset
Traning ratio	0.8	Train dataset ratio of the entire dataset
Optimizer	Adam	Optimization method for updating weights
Loss function	Cross-entropy	Loss function for training the model

be executed efficiently on CPUs without requiring GPU acceleration. However, it is important to note that traditional machine learning models like SVM and RF are generally limited to learning linear or relatively simple patterns. In contrast, deep learning models are capable of capturing more complex and nonlinear relationships in the data. Therefore, computational efficiency assessments typically focus on deep learning methods, where computational cost is a more critical concern.

Table S4: **Computational efficiency comparison.**

Mode	Metrics	DeepPROTACs	PROTAC-STAN	PROTAC-STAN (save attention map)	SVM	RF
Train	Time/epoch	9.23 seconds	5.9 seconds	-	0.47 seconds	0.4 seconds
	System memory	3374 MB	2918 MB	-	240 MB	235 MB
	GPU memory	1273 MB	3465 MB	-	-	-
Inference	Time/sample	0.568 seconds	0.508 seconds	0.536 seconds	0.02 seconds	0.02 seconds
	System memory	3165 MB	2812 MB	2821 MB	235 MB	235 MB
	GPU memory	1199 MB	913 MB	1269 MB	-	-

S1.2.3 Residue Selection and Position Assessment

In our analysis, we identified the **top 5** most highly weighted residues for the E3 ligase and POI as weighted residues, respectively. Specifically, we extracted residue weights from the ternary attention map by averaging across the POI and E3 ligase dimensions and normalizing the values to obtain residue-level attention scores. The top-weighted residues were then identified by ranking these scores and selecting the top five from each protein. Additionally, we included interaction residues involved in the 2D interaction diagrams from Maestro Simulation as interacting residues. Some residues, however, were unidentifiable due to sequence discrepancies between UniProt-derived protein sequences and experimentally determined structures from the PDB; these cases have been excluded accordingly.

To assess the position of residues in complex, we have computed the **distances of residues to the centroid of the PROTAC molecule**, with the results summarized in Table S5. 7KHH and 7JTP are examples in manuscript Section 2.3, and 6BOY are example in Supplementary Visualizations Section S1.4.4. This analysis provides a clearer view of the spatial distribution of these residues, showing that they are located within the PROTAC-binding pocket or close to the PROTAC molecule.

For two successful cases 7KHH and 6BOY, we can observe that the distances of residues are generally around 14 (± 4) Å, while the distance of interacting residues is around 10 Å. This indicates that most of the residues are close to the PROTAC molecule. In Complex 7KHH, in conjunction with Figure 5c (Article), it can be noted that residues with high weight, such as ASN-140, also overlap with interacting residues. In Complex 6BOY, TRP-81 and CYS-136, as interacting residues, also have high weights, which demonstrates the effectiveness of the interpretability of our model. Among the labeled residues, the proportion of high-weight residues in 7KHH reaches over half, and the proportion of high-weight residues in 6BOY reaches 75%.

For the failed case 7JTP, there is no overlap between weighted residues and interacting residues. Furthermore, based on the binding visualization and residue distance table, GLY-277 and ASN-281 among the 10 weighted residues are located further away from the PROTAC, with a distance exceeding 25 Å. However, the other 8 residues have a relatively similar distribution to the interacting residues and are located less than 25 Å from the PROTAC centroid. Although it failed, it shows the correct trend perception of important residues. This observation reflects a limitation of the current model: it is not explicitly designed for binding site prediction. Instead, the interpretability emerges as a by-product of the ternary attention mechanism, which learns to focus on informative regions for degradation prediction. While this does not ensure precise spatial alignment with all interacting atoms, the attention maps do offer biologically relevant and interpretable insights in many cases. We view this as a valuable and unexpected strength of our model, though we acknowledge the potential for future refinement—particularly by incorporating explicit binding site supervision or structural alignment metrics.

Table S5: **Residue weights, ranking and distance to the PROTAC centriod.** weighted: residues with weight from attention map, interacting: residues interact with PROTAC molecule from 2D interaction diagrams. 7KHH and 7JTP (manuscript Section 2.3), 6BOY (Supplementary Visualizations Section S1.4.4).

Complex	Location	Residue	Weight	Ranking	Distance (Å)	Role
7KHH	C:101	LEU-101	1.0000	1	18.58	weighted
	D:135	ASN-135	1.0000	1	15.89	weighted
	D:106	ASP-106	0.9900	3	15.77	weighted
	D:104	PRO-104	0.9721	4	17.65	weighted
	C:76	PHE-76	0.9115	5	15.60	weighted
	D:140	ASN-140	0.7889	6	11.04	interacting+weighted
	D:88	ASP-88	0.0710	7	9.46	interacting+weighted
	C:110	HIS-110	0.0000	8	9.37	interacting
	C:111	SER-111	0.0000	8	10.14	interacting
	C:115	HIS-115	0.0000	8	10.60	interacting
	C:98	TYR-98	0.0000	8	9.02	interacting
7JTP	L:101	LEU-101	1.0000	1	9.42	weighted
	A:277	GLY-277	1.0000	2	27.99	weighted
	A:147	GLY-147	0.9553	3	19.46	weighted
	A:151	GLU-151	0.9451	4	11.69	weighted
	L:76	PHE-76	0.9189	5	4.71	weighted
	A:281	ASN-281	0.8586	6	30.93	weighted
	A:232	ALA-232	0.8507	7	21.27	weighted
	A:105	ALA-105	0.8167	8	24.57	weighted
	A:130	ASN-130	0.8005	9	15.71	weighted
	A:88	LEU-88	0.7832	10	24.85	weighted
	L:98	TYR-98	0.0000	11	8.25	interacting
	L:110	HIS-110	0.0000	11	6.50	interacting
	L:111	SER-111	0.0000	11	6.85	interacting
	L:112	TYR-112	0.0000	11	10.32	interacting
	L:115	HIS-115	0.0000	11	11.55	interacting
	A:91	SER-91	0.0000	11	23.11	interacting
	A:107	ASP-107	0.0000	11	20.81	interacting
	A:133	PHE-133	0.0000	11	17.98	interacting
6BOY	C:135	ASN-135	1.0000	1	16.78	weighted
	C:106	ASP-106	0.9871	2	15.08	weighted
	C:104	PRO-104	0.9643	3	18.86	weighted
	B:49	PHE-49	0.9401	4	27.15	weighted
	C:81	TRP-81	0.7097	5	5.15	interacting+weighted
	C:136	CYS-136	0.6414	6	13.05	interacting+weighted
	B:378	HIS-378	0.0000	7	12.87	interacting
	B:380	TRP-380	0.0000	7	14.98	interacting

Moreover, we incorporated a quantitative spatial overlap evaluation to further validate the biological plausibility of the attention mechanism. Specifically, we calculated the average distance between each attention-weighted residue and its closest interacting residue. The resulting average distances were 6.04 Å for 7KHH, 15.42 Å for 7JTP, and 7.81 Å for 6BOY. We then assessed the proportion of high-attention residues located within a 10 Å threshold from any interacting residue. The percentages were 71.43% for 7KHH, 40% for 7JTP, and 66.67% for 6BOY. These results indicate substantial spatial overlap between high-attention residues and binding-site residues in 7KHH and 6BOY, supporting the interpretability of the model. The lower percentage in 7JTP is consistent with its higher average distance, likely reflecting weaker or less localized attention around functional interfaces in this specific case. Overall, these findings provide a more concrete and quantitative validation of the attention mechanism's alignment with biologically relevant regions.

S1.2.4 Top Residue Statistics

To support the findings in Figure 5 beyond Maestro simulations, we conducted an additional statistical analysis of amino acid residue distributions in active (labeled as "True") PROTAC samples from our dataset. Specifically, we performed frequency analysis on the protein sequences of POI and E3 ligases across all active samples. The top 10 most frequently occurring residues were identified as L, E, S, P, A, G, Q, K, V, and R. To assess the consistency between dataset-level residue importance and model attention, we further analyzed the residue-level attention weights for the example shown in Figure 5 (PDB IDs: 7KHH). For the protein, we computed the average attention weight per amino acid type. As shown in Table S6, for 7KHH, the top 10 high-weight residues were W, A, M, F, L, I, V, Y, E, and S. Notably, the intersection between these top-weight residues and the dataset-level top residues is L, S, E, A, V, indicating a strong overlap. This overlap supports the reliability of the attention mechanism in identifying meaningful residues.
Moreover, we calculated the Spearman's rank correlation between the model's high-attention residues' weights and the residues' frequency in the dataset. Using the top 10 high-attention residues from the protein 7KHH, we obtained a Spearman's ρ = –0.503 (p = 0.138), indicating a moderate correlation. We also evaluated the top 5 residues, yielding a stronger correlation of ρ = –0.700 (p = 0.188). While not statistically significant, these trends support the notion that the attention mechanism aligns with biologically meaningful residue patterns, thus reinforcing the model's interpretability.
Additionally, when comparing with the binding residues identified via Maestro simulation in Figure 5, we observe that key interacting residues such as SER-111 (S) and high-weight residues like LEU-101 (L) are both within the intersection set. This consistency between the Maestro-identified binding residues, model-derived high-weight residues, and statistically frequent residues in active samples provides strong corroboration for the accuracy of our residue-level interpretability.

Table S6: **Top residue statistics.**

PROTAC-fine	Residue	L	E	S	P	A	G	Q	K	V	R
	Count	64856	54027	52031	51980	45997	43476	41260	40652	38351	36554
7KHH	Residue	W	A	M	F	L	I	V	Y	E	S
	Weight	0.7839	0.7372	0.6892	0.6853	0.6703	0.6519	0.6120	0.5878	0.5693	0.5309

S1.2.5 Sequence Length Statistics of POI/E3 ligase

We also analyzed the length distribution of all proteins including POI and E3 ligase based on the Warhead table and E3 ligand table from PROTAC-DB. The results are as shown in Figure S2. The average length is 730, while the suggested length of the protein language model, i.e. ESM is 1022, which covers almost 80% of the proteins, so we adopt it as the maximum allowed sequence length.

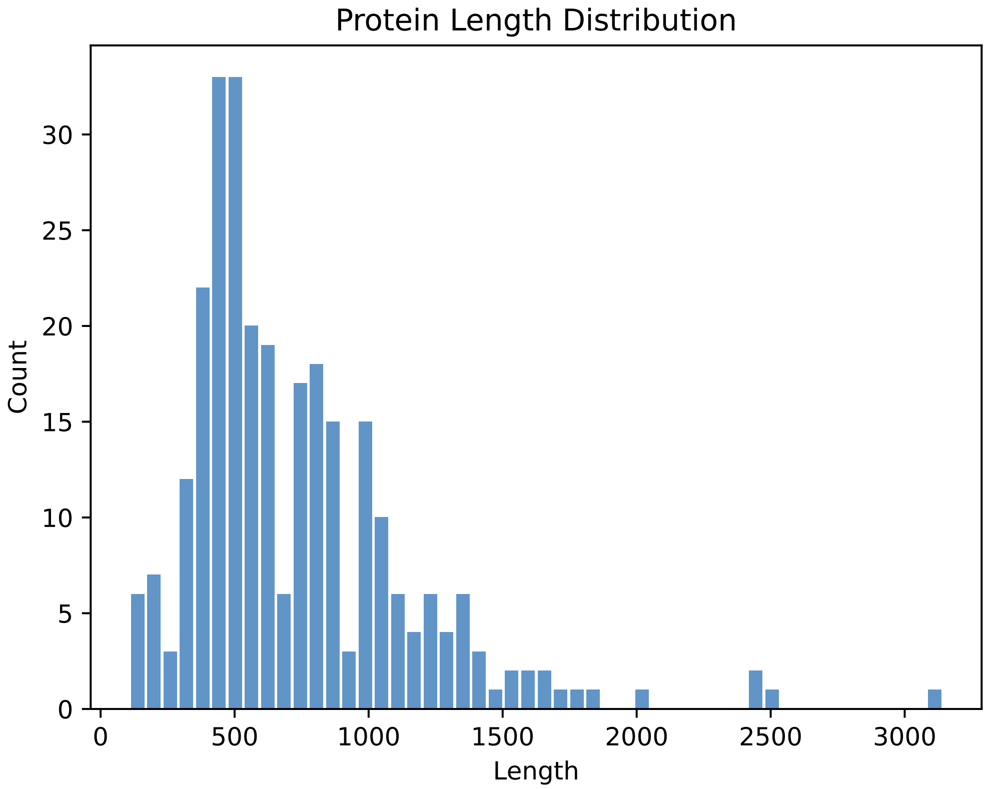

Figure S2: **Length distribution of POI/E3 ligase.**

S1.2.6 Molecular Properties in Global Feature

The molecular properties used in this study of PROTAC are shown in Table S7.

Table S7: **Molecular properties in global feature**

No.	Property
1	Molecular Weight
2	Exact Mass
3	LogP
4	Heavy Atom Count
5	Ring Count
6	Hydrogen Bond Acceptor Count
7	Hydrogen Bond Donor Count
8	Rotatable Bond Count
9	Topological Polar Surface Area (TPSA)

S1.2.7 Implicit DC_{50} and D_{max} from Experimental Descriptions

The explicit DC_{50} and D_{max} values in PROTAC-DB are relatively scarce. Upon thorough analysis of the PROTAC-DB data, we discovered that it contains partial descriptions of degradation experiments, as shown in Table S8. These primarily include *Percent degradation* and *Assay (Percent Degradation)*. By examining the Percent degradation at the corresponding Assay DC_{50} dose, it is possible to assess the degradation efficacy. Consequently, we can derive additional degradation activity information from these experimental descriptions.

Table S8: **Part of degradation experimental descriptions.**

Compound ID	Percent degradation (%)	Assay (Percent degradation)
99	8/29/65	% AR degradation in LNCaP cells after 6 h treatment at 10/100/1000 nM
373	76/>95/>99	% AR degradation in LNCaP cells after 6 h treatment at 10/100/1000 nM
427	90/94	% MEK1 degradation in A375 cells after 16 h treatment at 1000/10000 nM
428	91/96	% MEK1 degradation in A375 cells after 16 h treatment at 1000/10000 nM
456	0	% BCR-ABL degradation in K562 cells after 16 h treatment at 10000 nM
1163	45.6/77.3	% HDAC6 degradation in MM1S cells after 6 h treatment at 10/100 nM
1164	50.9/75.7	% HDAC6 degradation in MM1S cells after 6 h treatment at 10/100 nM
1480	<25/>=50/>=50	% KRAS degradation in H2023 cells at 300/1000/3000 nM
1481	<25/>=50/>=50	% KRAS degradation in H2023 cells at 300/1000/3000 nM
1493	>95/>95	% SHP2 degradation in KYSE520 cells after 6 h treatment at 100/1000 nM
1494	>95/>95	% SHP2 degradation in KYSE520 cells after 6 h treatment at 100/1000 nM
1697	30/56/95	% TPM3-TRKA degradation in KM12 cells after 6 h treatment at 0.1/1/10 nM
1698	22/28/86	% TPM3-TRKA degradation in KM12 cells after 6 h treatment at 0.1/1/10 nM
1806	37/71	% FAK degradation in PA1 cells after 8 h treatment at 1/10 nM
2029	97.1/95.9	% CDK6 degradation in MM.1S cells after 16 h treatment at 100/1000 nM
2039	25/82	% CDK6 degradation in MM.1S cells after 16 h treatment at 100/1000 nM
2113	>85/>85	% FAK degradation in PA-TU-8988T cells after 4 h treatment at 10/100 nM
2202	77.3/96.9	% ALK degradation in H3122 cells after 16 h treatment at 100/1000 nM
2210	77.4/85.9	% ALK degradation in Karpas 299 cells after 24 h treatment at 100/1000 nM
2626	69/72	% EGFR del19 degradation in HCC827 cells after 24 h treatment at 3/30 nM
2627	5.8/30.4	% SOS1 degradation in NCI-H358 cells after 24 h treatment at 100/1000 nM

S1.3 Ablation and Performance Analysis

S1.3.1 Component-wise Ablation on PROTAC-STAN

In our ablation study of PROTAC-STAN components, we initially constructed a basic model (Raw scheme) as a reference. The "-S1" experiment includes only the hierarchical PROTAC encoder to evaluate its impact, while "-S2" includes the structural protein encoder. The "-S" scheme includes both encoders simultaneously, allowing us to assess their coupled effect. By comparing "-S1" and "-S2" with the Raw baseline, we can analyze the individual contributions of each encoder; comparing "-S" with Raw reflects their joint contribution. An additional experiment that removes only the structural encoder, now referred to as "-S′", and presented the results in Table S9.
When comparing "-S′" and "-S1" with "Raw", we observe that the model performs better with the inclusion of the hierarchical encoder alone. This suggests that the hierarchical PROTAC encoder contributes more substantially to performance improvements. Notably, the performance gain is more prominent when using our proposed TAN fusion mechanism, indicating that our encoders are effectively capturing and integrating the relevant features.
While the contribution of the structural encoder appears smaller in isolation (as reflected by a smaller AUROC drop in "-S2"), this is partially due to the nature of the encoder inputs. The SMILESNet baseline only encodes SMILES sequences, whereas our hierarchical PROTAC encoder integrates SMILES, molecular properties, and graph-level features—resulting in a much richer representation. On the other hand, both the structural encoder and the N-gram encoder are based on protein sequences, and both produce latent protein embeddings, making their isolated effects appear more comparable.
Furthermore, the simple concatenation fusion strategy is less effective at capturing the deeper interactions among these encoded features, which may also explain the limited improvement observed when the structural encoder is used alone. However, comparing the full model ("-STAN") with "-S" demonstrates that our proposed TAN fusion mechanism significantly enhances performance by effectively integrating the three encoded feature types, including those from the structural encoder.

Table S9: **Ablation Study on PROTAC-STAN Components.** The Raw scheme is a base constructed from baselines, the -S1 and -S2 schemes ablate the PROTAC hierarchical and POI/E3 ligase structural encoders. The -S scheme combines -S1 and -S2 to assess feature encoding effects. The -S′ scheme removes only the structural encoder. The -T scheme evaluates the ternary attention network over concatenation. **Best**, <u>Second</u>.

Component	Raw	-S1	-S2	-S	-S′	-T	-STAN
SMILESNet encoder [3]	✓		✓			✓	
Hierarchical encoder		✓		✓	✓		✓
Ngrams encoder [4]	✓	✓			✓	✓	
Structural encoder			✓	✓			✓
Concatenation [3,4]	✓	✓	✓	✓			
TAN fusion					✓	✓	✓
Accuracy	81.64%	85.99%	81.64%	85.51%	<u>86.76%</u>	80.68%	**88.41%**
AUROC	0.8109	<u>0.8671</u>	0.8229	0.8631	0.8594	0.8108	**0.8833**
F1 score	0.7738	<u>0.8380</u>	0.7889	0.8333	0.8323	0.7753	**0.8588**

S1.3.2 SMILES Length and ESM-2 Ablation

Our choice of 128 as the maximum SMILES length primarily stems from considerations of computational efficiency, as powers of two (e.g., $128 = 2^7$) are commonly aligned with low-level operations such as memory management and GPU optimization. Although extending the SMILES length limit to 256 (2^8) also meets these computational considerations, our experimental results suggest that most essential molecular features are contained within the first 128 tokens. In addition, extra characters may introduce noise rather than provide meaningful information. To validate this point, we conducted an additional ablation study in which the SMILES length limit was increased to 256. As shown in Table S10, while

the 256-length model remains competitive, the 128-token model achieves better results on our current dataset. This outcome demonstrates that a maximum length of 128 can preserve representational completeness and computational efficiency while maintaining robust performance.
ESM-2 also captures structural information implicitly, making it a strong baseline for protein sequence modeling. Our choice of ESM-S[5] was motivated by its explicit structure-supervised task (remote homology detection), which enhances structural feature representation. This additional supervision can be particularly beneficial for structure-sensitive tasks. We have conducted this additional ablation study using ESM-2. The results in Table S10 indicate that while ESM-2 performs comparably well, ESM-S demonstrates a slight advantage, suggesting that explicit structural supervision provides additional benefits in this task. This structure-aware representation allows our model to effectively capture key structural information of proteins without requiring explicit 3D structure input, substantially expanding the model's applicability.

Table S10: **Ablation Results on the ESM-2 (Ablation 1) and PROTAC SMILES length (Ablation 2).**

Method	SMILES length	PLM	Accuracy	AUROC	F1 score
PROTAC-STAN	128	ESM-S	88.41%	0.8833	0.8588
Ablation 1	128	ESM-2	87.33%	0.8744	0.8471
Ablation 2	256	ESM-S	85.30%	0.8454	0.8222

S1.3.3 Prospective Integration of ESM-S with AlphaFold3

We implement a multi-step technical framework for ESM-S integration with AlphaFold3: For each POI-E3 pair, we first compute residue-level ESM-S embeddings to generate contact probability maps and interface propensity scores. We then use these ESM-S predictions to rank potential binding interfaces and filter low-likelihood interaction pairs, significantly reducing the computational burden for subsequent AF3 modeling. Additionally, we employ ESM-S to guide the selection of sequence regions enriched in interface contacts for targeted AF3 sampling, enabling more focused structure prediction. We further integrate the sampled AlphaFold3 structures with our existing framework models, ensuring efficient utilization of structural information through seamless data fusion and enhanced predictive accuracy.
This ESM-S/AF3 integration pipeline delivers several key benefits for PROTAC prediction: (1) we achieve enhanced confidence in ternary complex formation predictions through evolutionary sequence analysis that identifies favorable POI-E3 interaction interfaces, (2) we obtain substantial computational cost reduction by pre-filtering unlikely PROTAC candidates before resource-intensive AF3 modeling, (3) we improve predictive accuracy for PROTAC efficacy through the fusion of AF3-predicted structures with our framework models, enabling more precise degradation outcome predictions, and (4) we enhance cross-target predictive scalability for large-scale PROTAC screening through efficient structural sampling and robust prediction frameworks. The integration of AF3-predicted structures significantly improves our predictive capabilities by incorporating high-quality structural information into PROTAC activity predictions, leading to more reliable identification of successful degrader compounds.

S1.3.4 TAN Module Ablation Results

The ablation results of TAN module are demonstrated in Table S11.

S1.3.5 Degradation Performance: PROTAC-fine vs. PROTAC-DB 3.0

The reported accuracy of 88.41% corresponds to PROTAC-STAN trained and evaluated on the PROTAC-fine dataset using a train/test split, where the training set contains 1202 samples and the test set contains 207 samples. In contrast, the 75.42% accuracy was obtained by directly applying the model trained on PROTAC-fine to the full PROTAC-DB 3.0 dataset, which includes 3182 samples.

Table S11: **Ablation Study on TAN module.** This table compares the performance of concatenation, LMF, and TAN fusion methods, investigates the effects of multi-heads of TAN, TAN_1 for single head and TAN_2 for two heads, and explores the impact of varying orders of input features. **Best**, <u>Second</u>.

Method	PROTAC	E3 ligase	POI	Accuracy	AUROC	F1 score
Concatenation [3,4]	1st	2nd	3rd	85.51%	0.8631	0.8333
LMF [6]	1st	2nd	3rd	86.47%	0.8652	0.8372
PROTAC-STAN$_{pair}$	1st	2nd	3rd	85.02%	0.8571	0.8268
TAN_1	1st	2nd	3rd	85.02%	0.8531	0.8228
PROTAC-STAN (TAN_2)	1st	2nd	3rd	**88.41%**	**0.8833**	**0.8588**
TAN_2	1st	3rd	2nd	86.47%	0.8692	0.8409
TAN_2	2nd	1st	3rd	85.51%	0.8551	0.8256
TAN_2	2nd	3rd	1st	86.96%	0.8692	0.8421
TAN_2	3rd	1st	2nd	<u>87.44%</u>	<u>0.8772</u>	<u>0.8506</u>
TAN_2	3rd	2nd	1st	86.96%	0.8613	0.8344

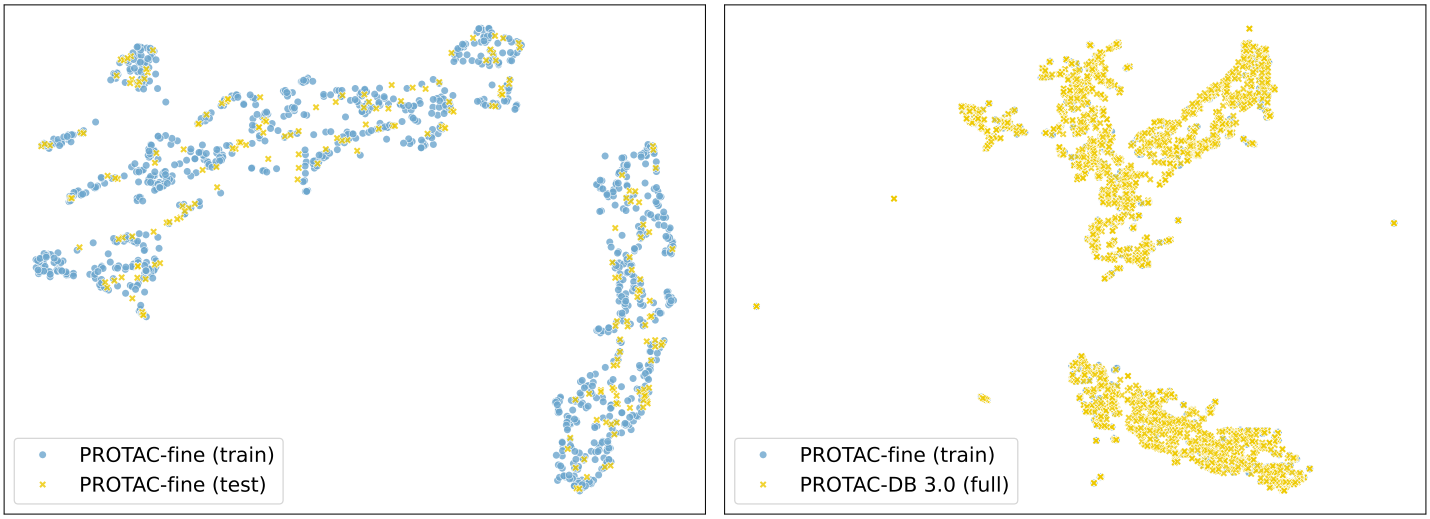

Figure S3: **UMAP distribution of PROTAC-fine (train), PROTAC-fine (test), and PROTAC-DB 3.0 (full).**

To investigate the cause of this performance drop, we conducted a distributional analysis using the data embeddings extracted from the first encoder layer of PROTAC-STAN. The sample distributions were visualized using UMAP, as shown in Figure S3. In the left panel, the PROTAC-fine training and test sets show similar distributions, indicating that the training and test samples are well-aligned in terms of class distribution and feature space. This suggests that the model generalizes well within the PROTAC-fine dataset.

However, the right panel reveals that the PROTAC-fine (train) set is only a subset of the full PROTAC-DB 3.0 dataset, which exhibits a broader and more complex distribution. This implies that PROTAC-DB 3.0 contains a more diverse or challenging set of molecules, likely including samples with distinct structural or functional characteristics not represented in the training data. This distributional shift may explain the observed decrease in generalization performance.

To further support this hypothesis, we conducted a KL divergence analysis. The KL divergence between PROTAC-fine (train) and PROTAC-fine (test) was 1.2, while the divergence between PROTAC-fine (train) and PROTAC-DB 3.0 (full) was 1.8—1.5 times higher. This confirms that there is a more substantial distribution shift between PROTAC-fine and PROTAC-DB 3.0, which likely contributes to the drop in prediction accuracy on the external dataset.

These results highlight the importance of training on more diverse and representative datasets for robust generalization, and we plan to incorporate broader data coverage in future model development.

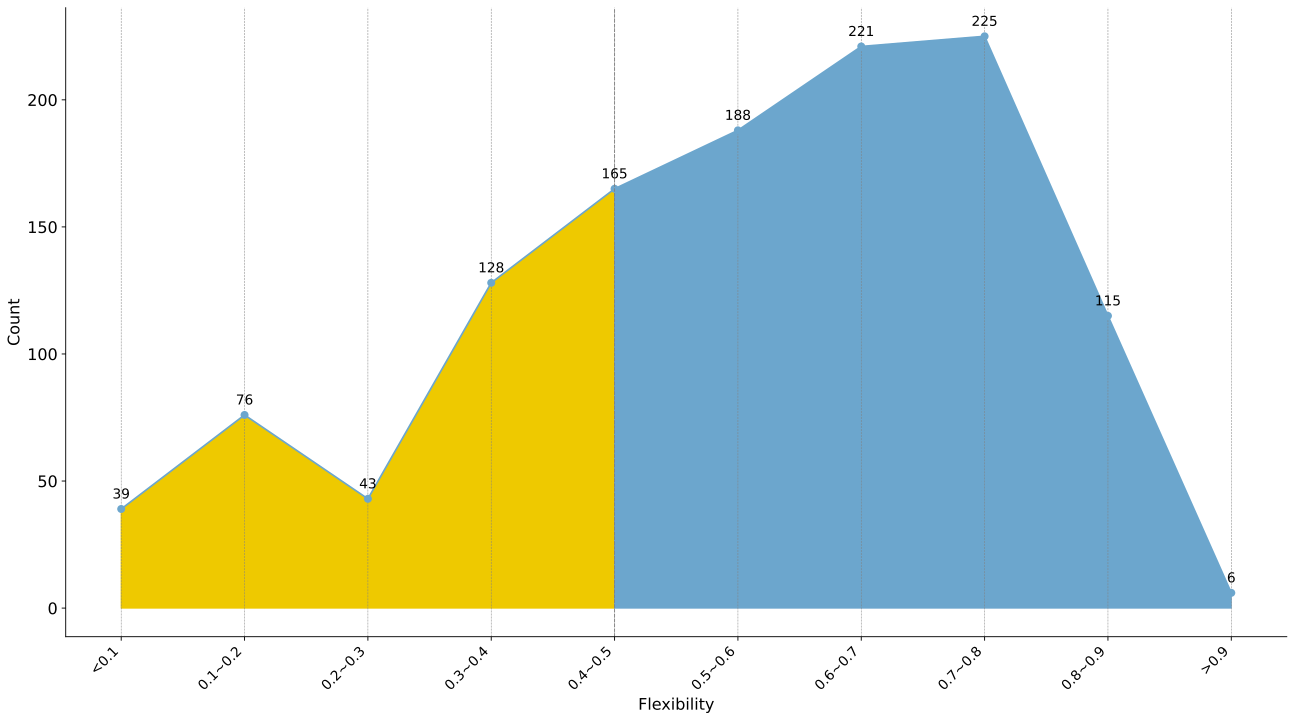

Figure S4: **Sample counts vs. linker flexibility across different linker flexibility groups.**

S1.3.6 Degradation Performance with Rigid Linkers

we performed statistical significance testing to evaluate whether the model exhibits biased performance between true and false samples in Figure 6a. We conducted Wilcoxon signed-rank tests[7] on the per-group prediction accuracy of true vs. false samples. The p-value across all groups was 0.2459, and after filtering out groups with fewer than 2 samples, the p-value remained non-significant at 0.4747. These results indicate that the model does not show a statistically significant difference in prediction accuracy between the two sample types, suggesting balanced performance.
Regarding the performance degradation observed for rigid linkers in Figure 6b, we categorized linkers into rigid and flexible based on a threshold of 0.5. We then analyzed their distribution, as shown in Figure S4. The analysis reveals an imbalance in the training dataset, with a higher proportion of flexible linkers. This imbalance also represents a current limitation of available PROTAC datasets. We believe that as these datasets become larger and more comprehensive—incorporating a wider variety of linker flexibilities—future models will be able to more accurately predict degradation across both rigid and flexible linker classes.

S1.4 Supplementary Visualizations

S1.4.1 3D Attention Map

Here, we present additional exemplars of 3D attention map visualization, leveraging three illustrative samples from PROTAC-DB 2.0 (Compound IDs: 339, 340, and 2286). Notably, as depicted in Figure S5, a consistent trend emerges as the above observation. Specifically, the leftmost two columns exhibit pronounced patterns, whereas the figures in the right final column demonstrate weaker patterns.

S1.4.2 2D Attention Map

We extend our analysis by presenting further 2D pairwise attention map visualizations as depicted in Figure S6, derived from the above 3D attention weights. Consistent with our previous observations, the visualizations reveal a notable distinction between the pairwise attention profiles. Specifically, the pairwise attention between PROTAC and E3 ligase, as well as between PROTAC and POI, exhibits rela-

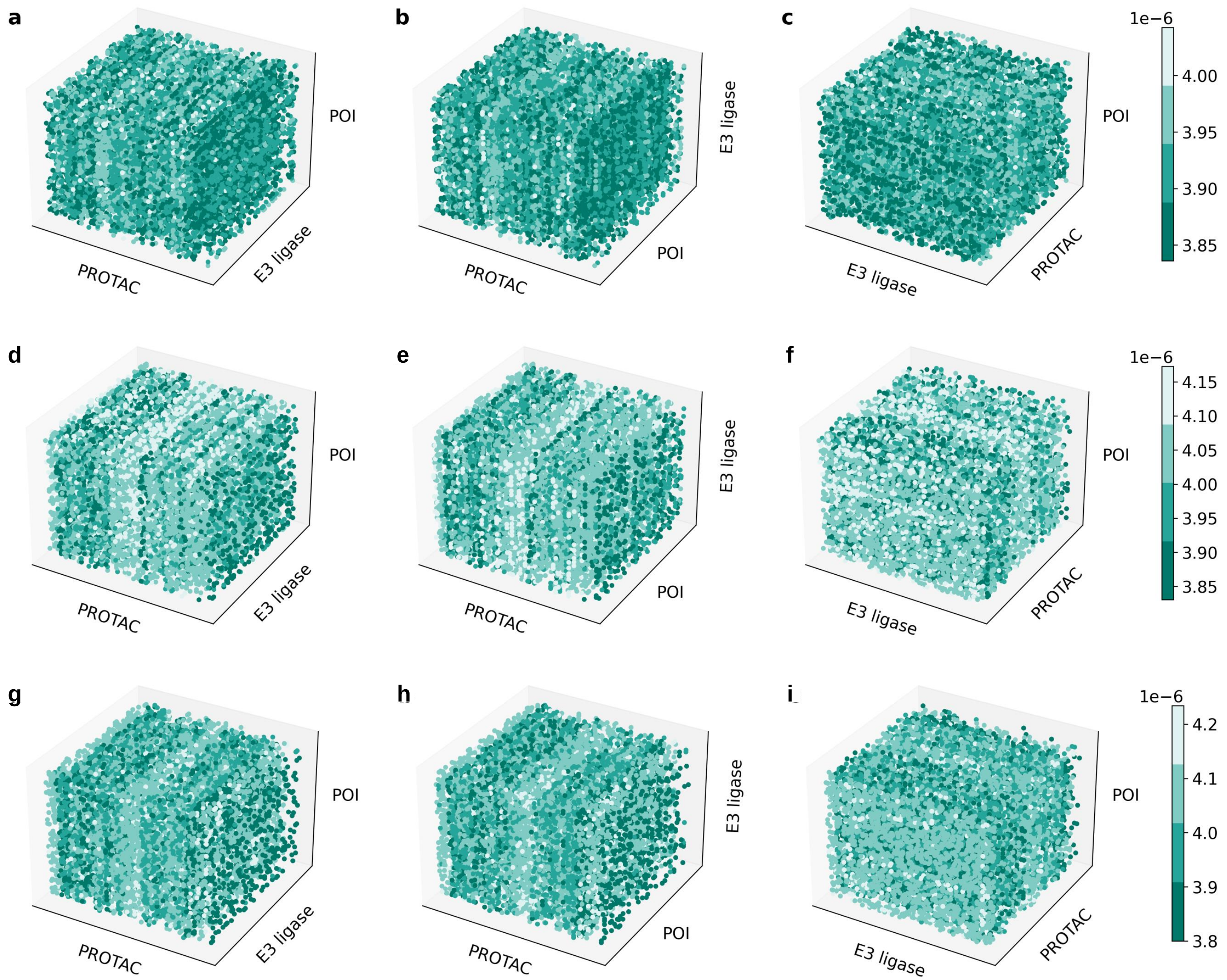

Figure S5: **More examples of 3D attention map visualization.** Sample 339 (a-c), sample 340 (d-f), and sample 2286 (g-i). Three figures in each row are front view, top view, and side view, respectively.

tively high values (deep blue-purple), whereas the pairwise attention between E3 ligase and POI display relatively lower values (medium yellow).

S1.4.3 2D PROTAC Molecule

We continue to map attention weights onto PROTAC molecules and 3D complexes. The visualized 2D PROTAC molecules with weighted atoms are demonstrated in Figure S7.

S1.4.4 2D Interaction and 3D Complex

The samples with 3D crystal structures are scarce. Consequently, we visualize one more example, providing 2D interaction and 3D complex visualization in comparison, as depicted in Figure S8.

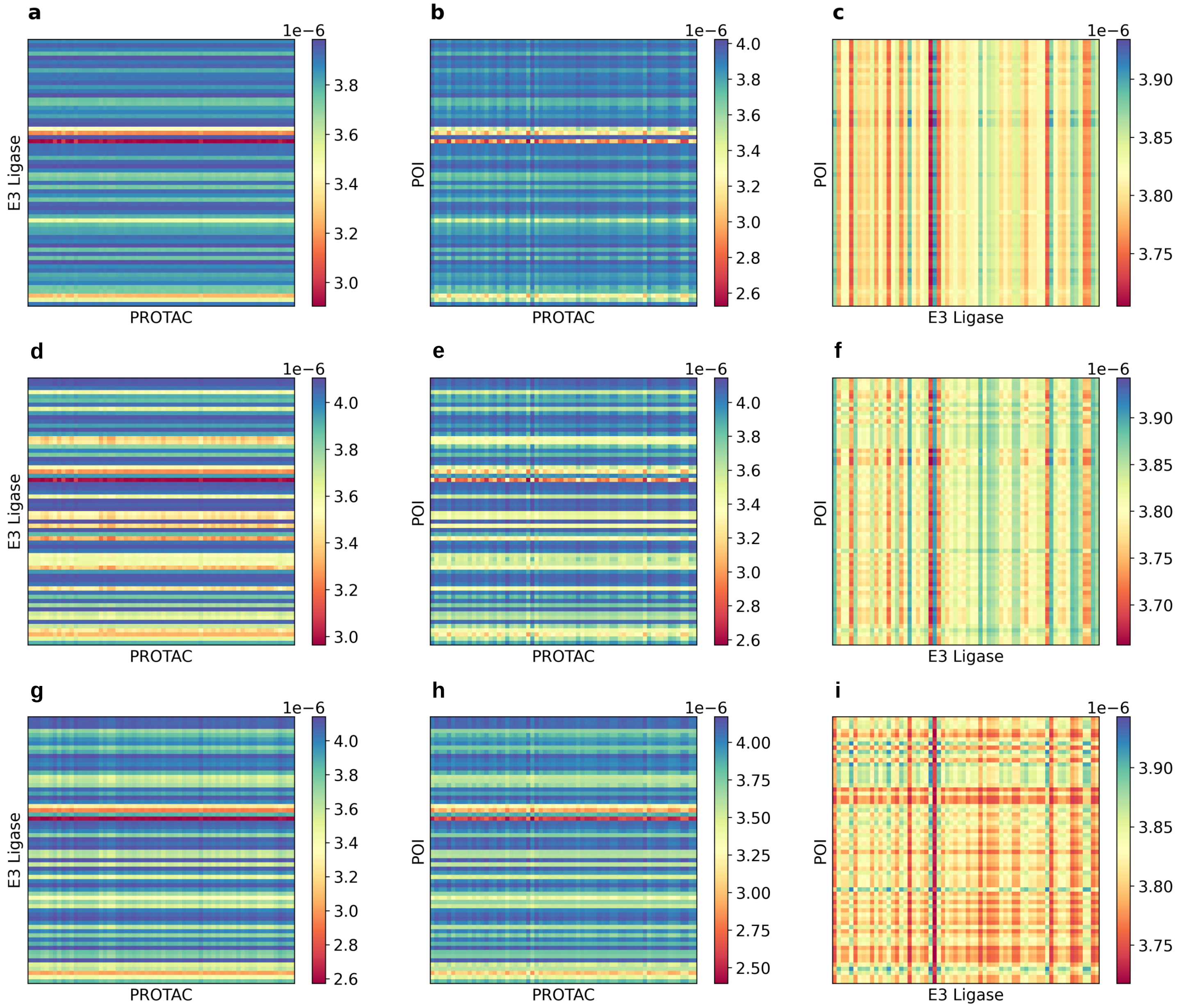

Figure S6: **More examples of 2D attention map visualization.** Sample 339 (a-c), sample 340 (d-f), and sample 2286 (g-i). Three figures in each row are pairwise attention maps of PROTAC-E3 ligase, PROTAC-POI, and E3 ligase-POI, respectively.

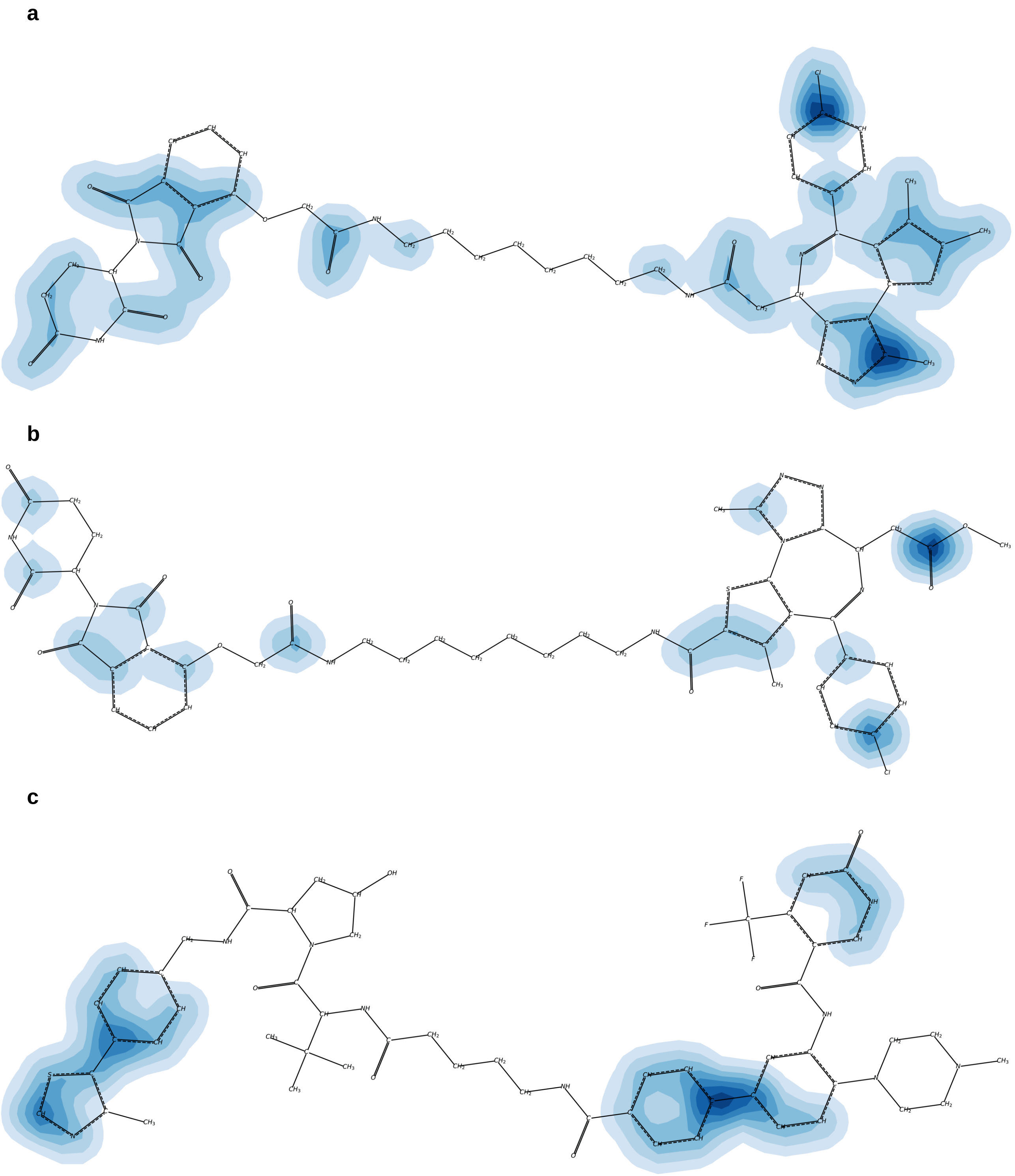

Figure S7: **More examples of weighted 2D PROTAC molecule. a.** Compound 339. **b.** Compound 340. **c.** Compound 2286.

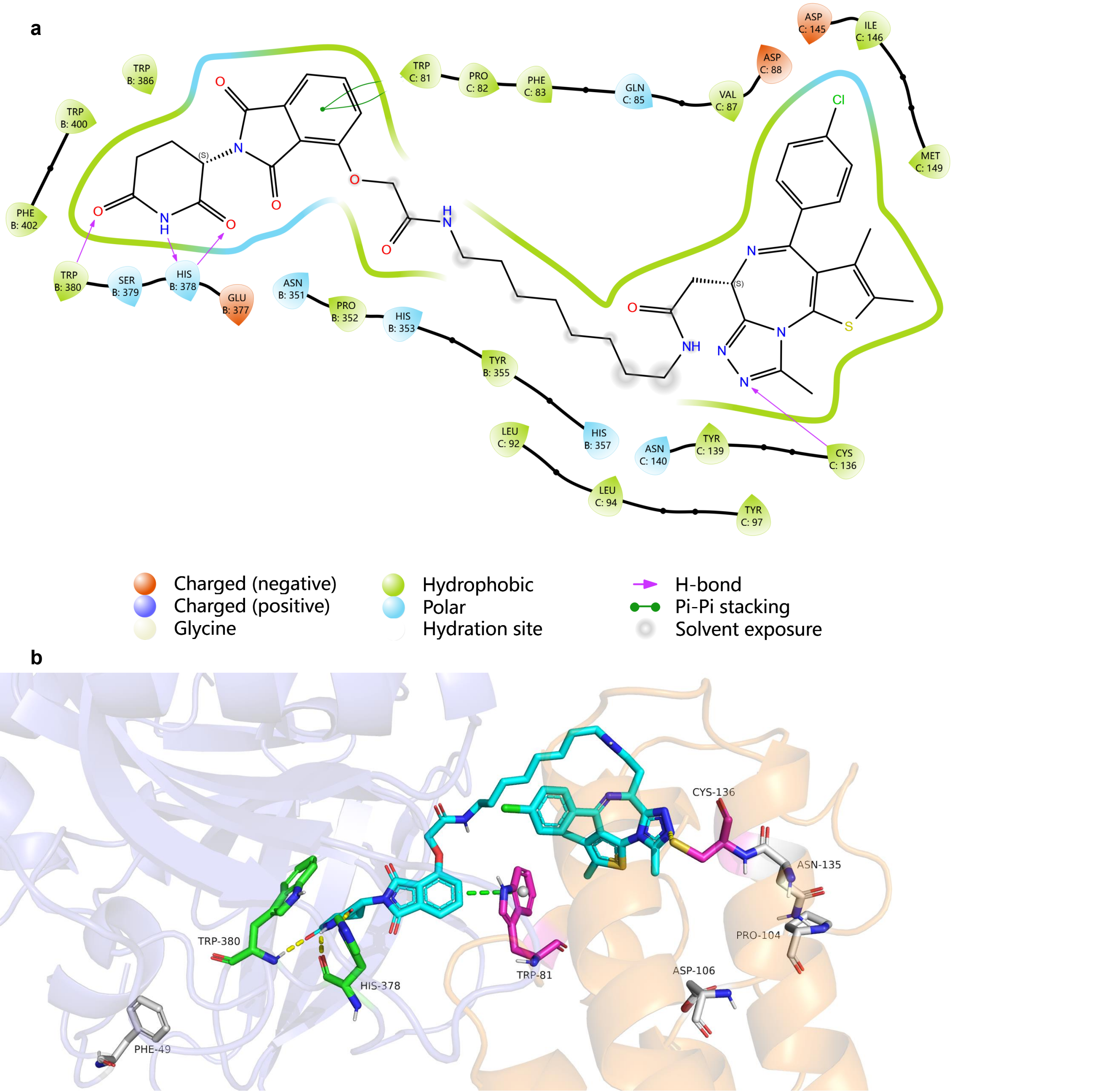

Figure S8: **2D interaction and 3D Complex visualization for example PDB 6BOY. (a)** 2D interactions of PROTAC and protein. There are mainly hydrogen bonding and π-π stacking interactions between PROTACs and proteins. **(b)** 3D pocket visualization. The top important weights in gray, and marked the actual interacting residues in green, overlapped residues in magenta, hydrogen bonds in yellow, and π-π stacking interactions in green. The blue-purple part in the background represents E3 ligase, while the orange part represents POI.

S2 Caption for Data S1

Ternary complexes (CDK4-PROTAC-VHL) for all 13 PROTACs. Data S1 includes the 13 novel PROTACs we constructed and their ternary complexes formed with CDK4 and VHL, saved in PDB format.

References

[1] A. Einstein, "Die Grundlage Der Allgemeinen Relativitätstheorie", *Annalen der Physik* **1916**, *354*, 7 769, https://doi.org/10.1002/andp.19163540702.

[2] J.-H. Kim, J. Jun, B.-T. Zhang, "Bilinear Attention Networks", **2018**, https://doi.org/10.48550/arXiv.1805.07932.

[3] F. Li, Q. Hu, X. Zhang, R. Sun, Z. Liu, S. Wu, S. Tian, X. Ma, Z. Dai, X. Yang, S. Gao, F. Bai, "DeepPROTACs Is a Deep Learning-Based Targeted Degradation Predictor for PROTACs", *Nature Communications* **2022**, *13*, 1 7133, https://doi.org/10.1038/s41467-022-34807-3.

[4] D. Nori, C. W. Coley, R. Mercado, "De Novo PROTAC Design Using Graph-Based Deep Generative Models", **2022**, https://doi.org/10.48550/arXiv.2211.02660.

[5] Z. Zhang, J. Lu, V. Chenthamarakshan, A. Lozano, P. Das, J. Tang, "Structure-Informed Protein Language Model", **2024**, https://doi.org/10.48550/arXiv.2402.05856.

[6] Z. Liu, Y. Shen, V. B. Lakshminarasimhan, P. P. Liang, A. Bagher Zadeh, L.-P. Morency, In I. Gurevych, Y. Miyao, editors, *Proceedings of the 56th Annual Meeting of the Association for Computational Linguistics (Volume 1: Long Papers)*. Melbourne, Australia, **2018** 2247–2256, https://doi.org/10.18653/v1/P18-1209.

[7] R. F. Woolson, "Wilcoxon Signed-Rank Test", In *Wiley Encyclopedia of Clinical Trials*, 1–3. John Wiley & Sons, Ltd, ISBN 978-0-471-46242-2, **2008**.
